# Dynamic domain arrangement of CheA-CheY complex regulates bacterial thermotaxis, as revealed by NMR

**DOI:** 10.1038/s41598-017-16755-x

**Published:** 2017-11-28

**Authors:** Yuichi Minato, Takumi Ueda, Asako Machiyama, Hideo Iwaï, Ichio Shimada

**Affiliations:** 1 Graduate School of Pharmaceutical Sciences, The University of Tokyo, Tokyo, Japan; 2 Precursory Research for Embryonic Science and Technology, Japan Science and Technology Agency, Kawaguchi, Japan; 3 Research Program in Structural Biology and Biophysics, Institute of Biotechnology, University of Helsinki, Helsinki, Finland

## Abstract

Bacteria utilize thermotaxis signal transduction proteins, including CheA, and CheY, to switch the direction of the cell movement. However, the thermally responsive machinery enabling warm-seeking behavior has not been identified. Here we examined the effects of temperature on the structure and dynamics of the full-length CheA and CheY complex, by NMR. Our studies revealed that the CheA-CheY complex exists in equilibrium between multiple states, including one state that is preferable for the autophosphorylation of CheA, and another state that is preferable for the phosphotransfer from CheA to CheY. With increasing temperature, the equilibrium shifts toward the latter state. The temperature-dependent population shift of the dynamic domain arrangement of the CheA-CheY complex induced changes in the concentrations of phosphorylated CheY that are comparable to those induced by chemical attractants or repellents. Therefore, the dynamic domain arrangement of the CheA-CheY complex functions as the primary thermally responsive machinery in warm-seeking behavior.

## Introduction

Cells utilize various systems for rapidly sensing the external cellular environment and adapting to environmental changes. Bacteria sense temperature and extrernal cellular chemical attractants or repellents, and migrate by changing the exchange rate of the direction of flagellar rotation. In thermotaxis, the direction of cell movement reportedly switches between warm-seeking and cold-seeking, to accumulate at the preferred temperature (~310 K)^1^. Bacterial chemotaxis and thermotaxis are regulated by the same two-component signal transduction system^1–8^. In this system, the signal transduction from transmembrane chemoreceptors, which bind to the external cellular attractants and repellents, to the flagellar motors is mediated by CheA and CheY. CheA is a 154 kDa dimeric protein composed of the P1, P2, P3, P4, and P5 domains, and CheY is a 14 kDa single-domain protein (Supplementary Fig. S1a)^9,10^. A histidine residue on the P1 domain (H48 in *Escherichia coli*) is first autophosphorylated by the P4 domain, which binds to ATP^11–17^. The phosphoryl group on the P1 domain is subsequently transferred to an aspartic acid of CheY (D57 in *E. coli*), which forms a complex with the P2 domain^18–22^ (Supplementary Fig. S1b). The phosphorylated CheY increases the exchange rate of the direction of flagellar rotation, by binding to the switch protein FliM.

In chemotaxis, the sensor of the attractants and repellents is the periplasmic region of the chemoreceptor: the attractants and repellents bind to the chemoreceptor-CheA complex and decrease and increase the autophosphorylation rate of CheA, respectively, thus regulating the concentration of phosphorylated CheY. However, in thermotaxis, the temperature-dependent modulation of the conformation of the chemoreceptor trimers was not observed at temperatures below 303 K^23^, at which wild type cells move to the warmer direction, although the ~40 kJ/mol activation energy of the aforementioned overall CheY phosphorylation reaction, which is larger than that of the dephosphorylation, indicated that the thermotaxis is regulated by the temperature-dependent change of the CheY phosphorylation rate^24^.

The three-dimensional structures of CheY^25,26^, the isolated P1 and P2 domains^27–29^, and their complexes^30–33^ have been solved, and the binding interfaces on the P1 and P2 domains of CheA and CheY have been identified (Supplementary Fig. S1c and d). In addition, NMR spectra of full-length CheA were previously reported^34,35^. However, these structures did not provide clues for the elucidation of the mechanism underlying the temperature-dependent change of the CheY phosphorylation rate. Therefore, the effects of temperature on the structures and dynamics of the bacterial chemotaxis/thermotaxis signaling proteins and their relevance to the overall thermotaxis signaling must be clarified.

Here, we used NMR, along with the quantitative time-course simulation of the signaling, to investigate the effect of temperature on the structure and dynamics of the full-length CheA and CheY complex, and discussed the mechanism of thermotaxis.

## Results

### The CheA-CheY complex exists in equilibrium between multiple states with various domain arrangements

In the ^1^H-^15^N TROSY spectrum of [^2^H, ^15^N] CheA from *Escherichia coli*, resonances with chemical shifts almost identical to those of the isolated P1 and P2 domains were observed, whereas most of the resonances from the P3, P4, and P5 domains were not detected (Supplementary Fig. S2a). The total correlation times of the P1 and P2 domains, determined by transverse relaxation-optimized spectroscopy for rotational correlation times (TRACT) experiments^36^, were ~30 and ~15 ns, respectively. These values are remarkably smaller than those expected from the size of CheA (68 ns), suggesting that the P1 and P2 domains are structurally isolated from the other domains by flexible linkers (see Supplementary Note and Supplementary Fig. S2 for the resonance assignments). The spectra were significantly simplified by the segmental labeling of CheA^35^. In the spectra of [^2^H,^15^N]-P1/P2–5 CheA and [u-^2^H, Ileδ1-^13^CH_3_, Metε-^13^CH_3_, Alaβ-^13^CH_3_]-P1/[^2^H]-P2–5 CheA, in which the P1 domain of CheA was segmentally labeled, resonances from the P1 domain of CheA were selectively observed (Supplementary Fig. S2).

**Figure 1:**
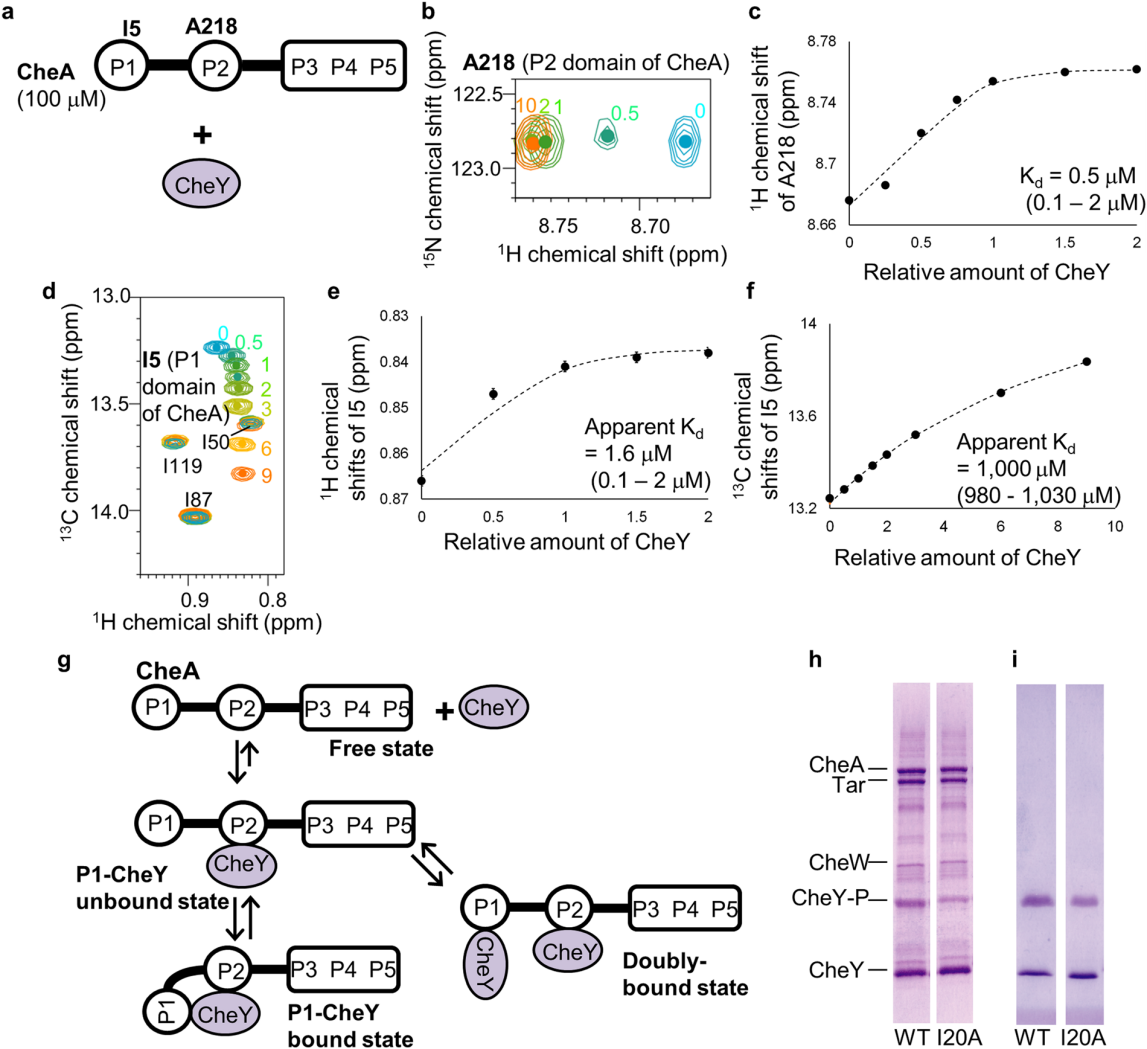
Interaction modes of CheA and CheY revealed by titration experiments. (**a**) Schematic diagram of the experiment. (**b**) Overlaid ^1^H-^15^N TROSY spectra of [^2^H, ^15^N] CheA in the presence of various amounts of non-labeled CheY. Only the regions with A218 resonances are shown, and the amounts of CheY relative to CheA are indicated. Full spectra in the absence of CheY are shown in Supplementary Fig. S2. (**c**) Plots of the ^1^H chemical shifts of the resonances from A218 against the relative amount of CheY. (**d**) Overlaid ^1^H-^13^C HMQC spectra of the [u-^2^H, Ileδ1-^13^CH_3_, Metε-^13^CH_3_, Alaβ-^13^CH_3_]-P1/[^2^H]-P2–5 CheA in the presence of various amounts of non-labeled CheY, observed at 303 K. Only the regions with the I5, I50, I87, and I119 resonances are shown. The centers of the I5 resonances are indicated with dots, and the amounts of CheY relative to CheA are indicated. (**e**,**f**) Plots of the ^1^H (**e**) and ^13^C (**f**) chemical shifts of the resonances from I5, observed at 303 K, against the relative amounts of CheY. In (**c**,**e** and **f**), the apparent dissociation constants were determined by non-linear least squares fitting (dotted curves), using the Levenberg–Marquardt algorithm and the reported equation of the 1:1 binding model^85^. Error bars represent standard deviations of the chemical shifts of 100 synthetic time domain data from the *in situ* error analysis^86^. (**g**) Schematic diagrams of the binding modes. (**h**,**i**) Phosphorylation of CheY (left) or CheY/I20A (right) by CheA and its activators CheW and Tar (**h**) or acetyl phosphate (**i**), detected by phosphate-affinity SDS-PAGE. The bands of the phosphorylated CheY, CheY-P, are indicated, as well as those of CheA, Tar, CheW, and CheY. In (**h**,**i**), images are cropped from different parts of the same gel, and the full length gel images are shown in Supplementary Fig. S5.

In order to observe the interactions of CheY with the P1 and P2 domains of CheA, which reportedly exhibit low and high affinities for CheY, respectively^33^, NMR spectra of 100 μM uniformly or segmentally labeled CheA were recorded in the presence of various concentrations of non-labeled CheY (Fig. 1a). The resonances from residues on the P2 domain of CheA, such as A218, linearly shifted by increasing the concentration of CheY, until the molar ratio of CheA and CheY became ~1:1 (Fig. 1b), and the dissociation constant (K_d_) was calculated as 0.5 μM (0.1 ~ 2 μM) by fitting a 1:1 binding model to the data (Fig. 1c). This dissociation constant is similar to those previously reported for the isolated P2 domain^21^, indicating that the chemical shift perturbation reflects the interaction between the P2 domain of CheA and CheY. The resonances from the residues on the P1 domain of CheA shifted non-linearly upon the addition of CheY. For example, the resonance from I5 of CheA exhibited a ^1^H upfield shift upon the addition of a stoichiometric amount of CheY, and interestingly exhibited a remarkable ^13^C downfield shift upon the further addition of excess amounts of CheY (Fig. 1d–f). We confirmed that the chemical shift perturbations observed for the P1 domain of CheA upon the addition of CheY reflected the direct interaction between the P1 domain of CheA and CheY (Supplementary Note, Supplementary Figs S3 and 4a–c, and Supplementary Table S1 ^37^).

The dissociation constant, calculated by fitting the 1:1 binding model to the ^1^H chemical shifts observed for I5 of CheA, was 1.6 μM (0.1–2 μM) (Fig. 1e). Thus the apparent affinity of the P1 domain of CheA for CheY was remarkably higher than that of the isolated P1 domain (dissociation constant = 900 μM (700-1,100 μM), Supplementary Note and Supplementary Fig. S4d–g) and comparable to that of the P2 domain of CheA (K_d_ = 0.5 μM (0.1 ~ 2 μM), Fig. 1c). This high affinity interaction between the P1 domain of CheA and CheY, which is comparable to that between the P2 domain of CheA and CheY, suggested that the P1 and P2 domains in a single CheA molecule could simultaneously bind to a single CheY molecule. Hereafter, the state in which the P1 and P2 domains in a single CheA molecule simultaneously bind to a single CheY molecule is referred to as the “P1-CheY bound state” (Fig. 1g).

The ^13^C chemical shifts of I5 in CheA were perturbed upon the addition of excess amounts of CheY (Fig. 1f), whereas > 80% of the P2 domain of CheA bound to CheY upon the addition of a stoichiometric amount of CheY (Fig. 1c). Therefore, the ^13^C chemical shift perturbations of I5 in CheA correspond to the binding of another CheY molecule to the CheA-CheY complex. Hereafter, the state in which two CheY molecules bind to a CheA molecule is referred to as the “doubly-bound state” (Fig. 1g).

In order to examine the CheA-CheY binding modes in the P1-CheY bound state and the doubly-bound state, we searched for conditions in which either the P1-CheY bound state or the doubly-bound state is predominantly observed. The apparent dissociation constants of the P1-CheY bound state and the doubly-bound state are 1.6 μM and 1,000 μM, respectively, as calculated from the resonances from I5 of CheA (Fig. 1e,f). Using these apparent dissociation constants, we calculated the populations of the P1-CheY bound state and the doubly-bound state in the aforementioned titration experiments. Our calculations revealed that the equilibrium between the P1-CheY bound state and the doubly-bound state significantly shifts toward the former and latter states, under the conditions with 0.1 mM and 0.9 mM CheY, respectively. The chemical shift perturbations observed for the P1 domain of CheA upon the addition of 0.1 mM and 0.9 mM CheY, which predominantly reflect the P1-CheY bound state and the doubly-bound state, respectively, are plotted in Supplementary Fig. S4h,i. The residues that exhibited chemical shift perturbations in the P1-CheY bound state remarkably overlapped with those in the doubly-bound state (Supplementary Fig. S4h,i ^38^), suggesting that the P1-CheY bound state and the doubly-bound state share the CheY-binding site on the P1 domain of CheA. Therefore, to form the doubly-bound state, the P1 domain of CheA in the P1-CheY bound state dissociates from CheY, leading to the formation of the state in which the P1 domain of CheA dissociates from CheY and only the P2 domain of CheA binds to CheY (Fig. 1g). Hereafter, the state in which the P1 domain of CheA dissociates from CheY and only the P2 domain of CheA binds to CheY is referred to as the “P1-CheY unbound state”.

In *E. coli*, the physiological CheA and CheY concentrations are reportedly ~10 μM^39^. At these concentrations, CheA primarily exists in equilibrium between the P1-CheA unbound state and the P1-CheA bound state, and the doubly-bound state, which is the indirect evidence for the P1-CheY unbound state, is non-physiological.

In order to examine the effects of the dynamic domain arrangement of the CheA-CheY complex on the signaling, an *in vitro* signaling assay was performed using the CheY/I20A mutant, in which the P1 domain-CheY interaction of the CheA-CheY complex was disrupted (Supplementary Note, Supplementary Figs S3 and S4a–c, and Supplementary Table S1). In the presence of ATP, CheY and CheY/I20A were phosphorylated by CheA, which is activated by the receptor protein Tar and the adaptor protein CheW. The phosphorylated and unphosphorylated CheY and CheY/I20A were analyzed by phos-tag^TM^ SDS-PAGE. As a result, the band intensity of the phosphorylated CheY/I20A was remarkably lower than that of the phosphorylated CheY, and our densitometric analyses of the bands corresponding to the phosphorylated and unphosphorylated CheY and CheY/I20A indicated that the population of the phosphorylated CheY was < 50% of that of the wild type (Fig. 1h and Supplementary Fig. S5). Both CheY and CheY/I20A were phosphorylated by acetyl phosphate, which reportedly directly phosphorylates the D57 residue of CheY without utilizing the P1 domain binding site^40^ (Fig. 1i), suggesting that the reactivity of D57 was not perturbed by the I20A mutation. Therefore, the disruption of the dynamic domain arrangement of the CheA-CheY complex by the I20A mutation decreased the population of the phosphorylated CheY in the *in vitro* signaling assay.

### Equilibrium shifts toward the P1-CheY bound state with increasing temperature

**Figure 2:**
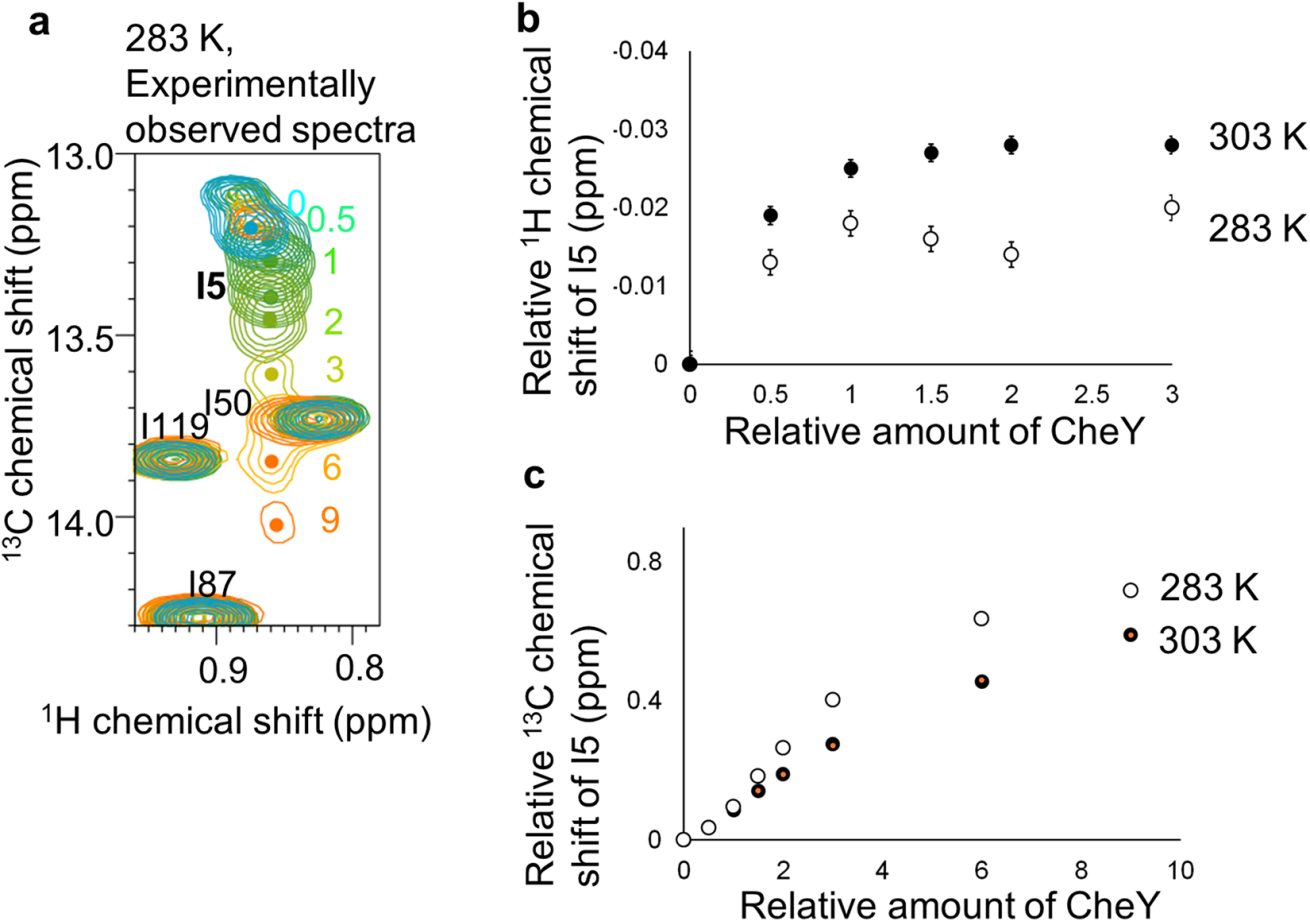
Effects of temperature on the interaction mode of CheA and CheY. (**a**) Overlaid ^1^H-^13^C HMQC spectra of the [u-^2^H, Ileδ1-^13^CH_3_, Metε-^13^CH_3_, Alaβ-^13^CH_3_]-P1/[^2^H]-P2–5 CheA in the presence of various amounts of non-labeled CheY, observed at 283 K. Only the regions with the I5, I50, I87, and I119 resonances are shown. The centers of the I5 resonances are indicated with dots, and the amounts of CheY relative to CheA are indicated. (**b**,**c**) Plots of the ^1^H (**b**) and ^13^C (**c**) chemical shifts of the resonances from I5, observed at 303 K (black circles, shown in Fig. 1e,f) or 283 K (white circles), against the relative amounts of CheY. Error bars represent standard deviations of the chemical shifts of 100 synthetic time domain data from the *in situ* error analysis^84^.

To examine the effect of temperature on the equilibrium of the CheA-CheY complex, we recorded the spectra of a 100 μM solution of segmentally labeled CheA in the presence of various concentrations of non-labeled CheY, at a lower temperature, 283 K (Fig. 2a). As a result, the ^1^H chemical shift perturbations observed for I5, which predominantly reflect the P1-CheY bound state, were smaller than those at 303 K (Figs 1d,e and 2a,b). These results suggested that the CheA-CheY complex exists in equilibrium between the P1-CheY bound state and the P1-CheY unbound state, with exchange rates faster than the NMR timescale (>1,000 s^−1^), and the equilibrium shifted toward the P1-CheY unbound state at 283 K. The ^13^C chemical shift perturbations, which predominantly reflect the doubly-bound state, were larger than those at 303 K (Figs 1d,f, 2a,c), suggesting that the population of the doubly-bound state increased with a decrease in the temperature.

**Figure 3:**
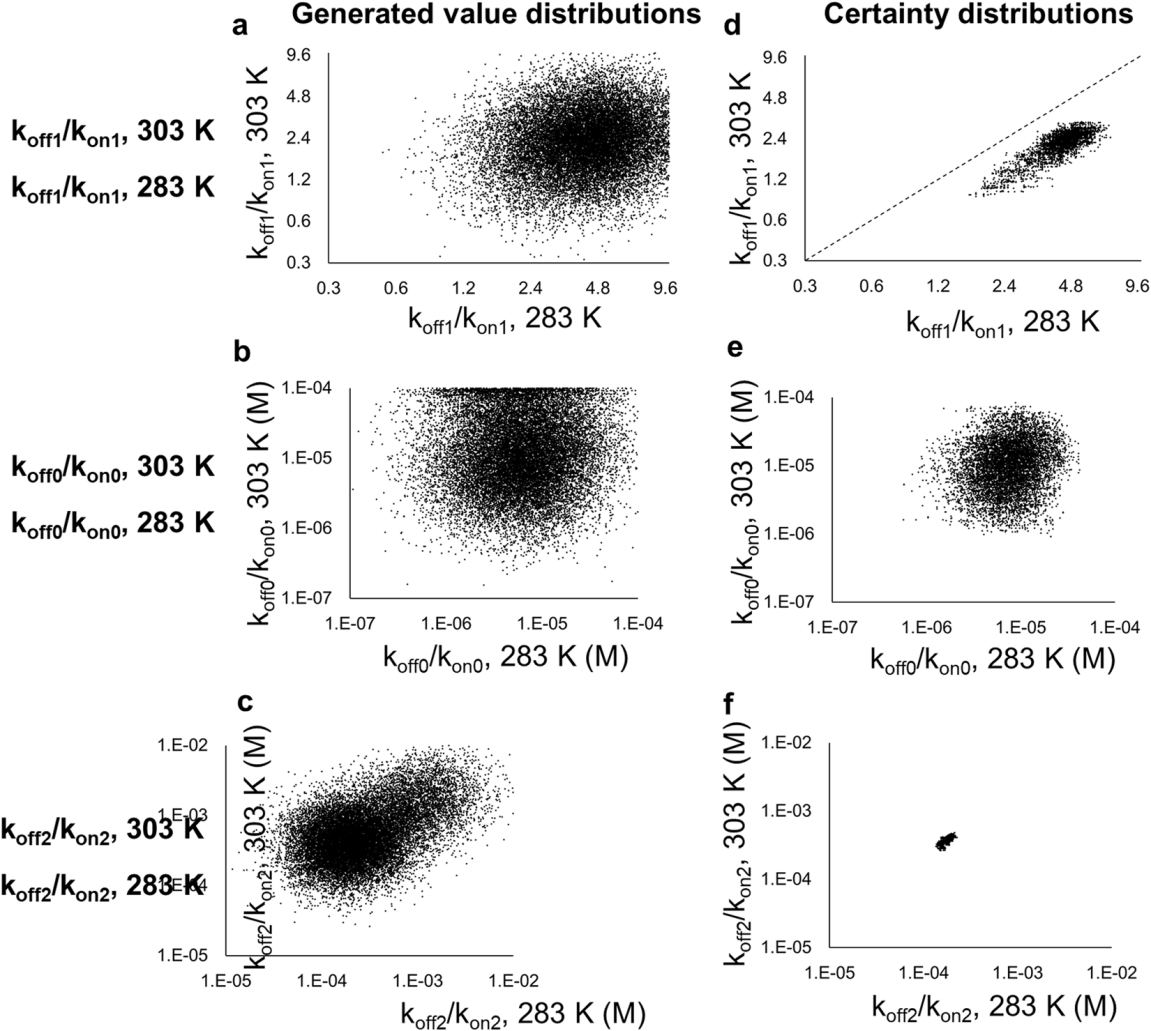
Generated value distributions and certainty distributions of the equilibrium constants of the dynamic domain arrangement of the CheA-CheY complex. (**a**–**c**) Plots of the generated k_off1_/k_on1_ (**a**), k_off0_/k_on0_ (**b**), and k_off2_/k_on2_ (**d**) at 303 K against those at 283 K. (**d**–**f**) Plots of the certainty distributions of k_off1_/k_on1_ (**d**), k_off0_/k_on0_ (**e**), and k_off2_/k_on2_ (**f**) at 303 K against those at 283 K. In (**d**–**f**), data during the burn-in period were excluded from the plots. At the dotted line in (**d**), k_off1_/k_on1_ at 303 K equals that at 283 K.

**Figure 4:**
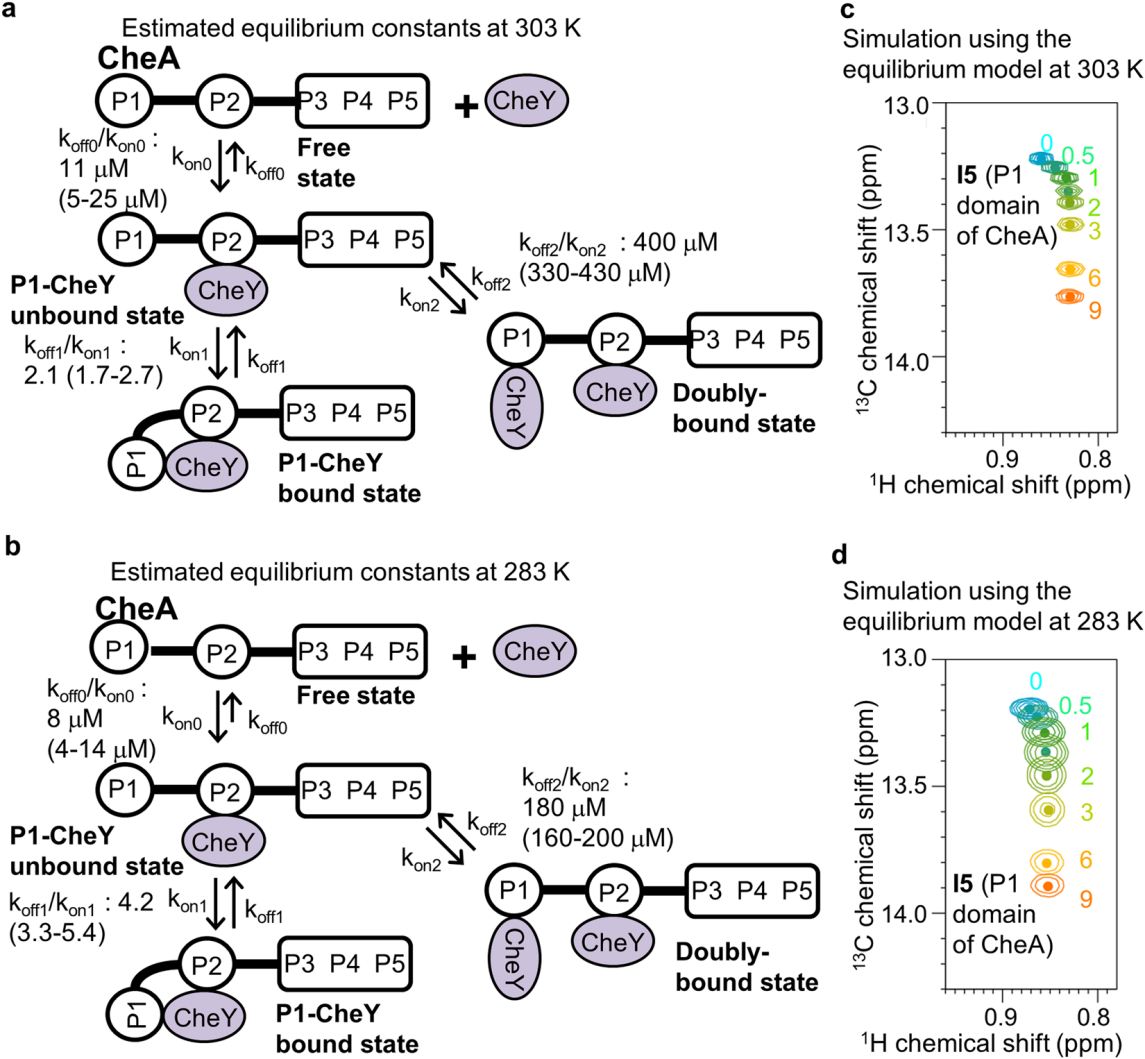
Temperature-dependent shift of the equilibrium of the CheA-CheY complex. (**a**,**b**) Estimated equilibrium constants and their standard deviations at 303 K (**a**) and 283 K (**b**), determined by the Markov chain Monte Carlo algorithm. (**c**,**d**) are overlaid resonances from the methyl group of I5 of CheA, in the presence of various concentrations of CheY, simulated using the equilibrium constants in (**a**,**b**), respectively, with the same colors as in Figs 1d and 2a.

In order to investigate the equilibrium in detail, we performed the simulations of the I5 resonances at 283 K and 303 K, by using the model in Fig. 1g. In the simulations, we applied the Bayesian inference that uses Markov chain Monte Carlo (MCMC) algorithms, which has been utilized in several biomolecular NMR studies^41–44^ and has gained popularity in many fields including astrophysics, systems biology, and econometrics^45^. This method randomly generates numerous parameter sets, and the differences between the data calculated from each parameter set and the observed data are calculated. The differences are utilized to obtain the distribution of the certainty in the parameter space, which enables the determination of the uniqueness of the estimates. Although the parameter space of the model in Fig. 1g is huge, the MCMC algorithms reportedly allow us to consider the entire parameter space for arbitrary complicated models^45–47^. The plots of the generated parameter sets and the probability distribution plots, in which the certainties are represented as the density of the points, are shown in Fig. 3 and Supplementary Fig. S6, and the equilibrium constants with the highest certainty and the simulated I5 signals at 303 K and 283 K are shown in Fig. 4. The distributions of the generated parameters were remarkably larger than the certainty distributions, and each certainty distribution formed a cluster, indicating the uniqueness of the solution (Fig. 3 and Supplementary Fig. S6). The certainty distribution of k_off1_/k_on1_ at 303 K and 283 K exists in the region where k_off1_/k_on1_ at 283 K is larger than that at 303 K (Fig. 3d), suggesting that the equilibrium between the P1-CheY unbound state and the P1-CheY bound state shifted toward the former at a lower temperature. The resonances generated from the constants with highest certainty (Fig. 4c,d) were in good agreement with the observed resonances (Figs 1d and 2a).

**Figure 5:**
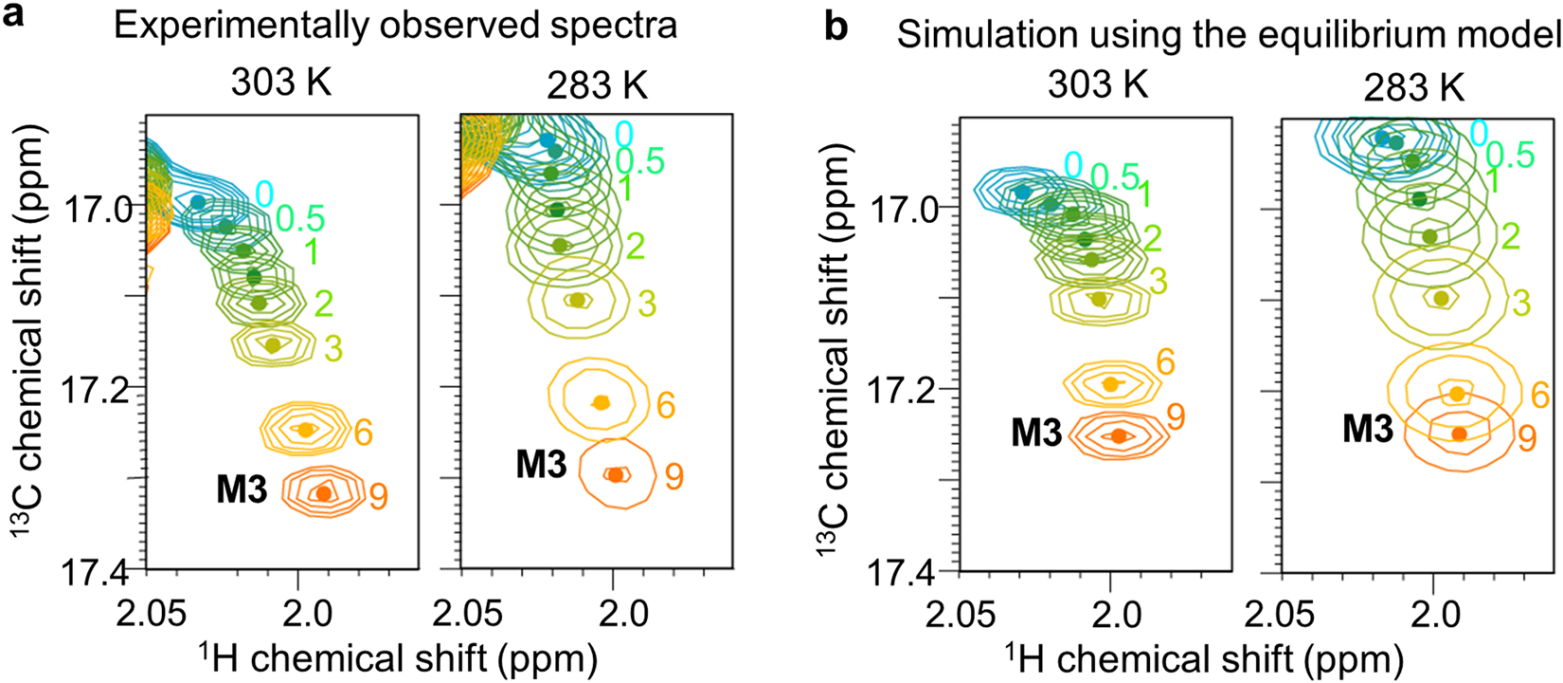
Non-linear shift of the resonances from M3. (**a**) Overlaid ^1^H-^13^C HMQC spectra of [u-^2^H, Ileδ_1_-^13^CH_3_, Metε-^13^CH_3_, Alaβ-^13^CH_3_]-P1/[^2^H]-P2–5 CheA in the presence of various amounts of non-labeled CheY, observed at 303 K (left panel) and 283 K (right panel). Only the regions with the M3 resonances are shown. The centers of the M3 resonances are indicated with dots, and the amounts of CheY relative to CheA are indicated. (**b**) Overlaid resonances from the methyl group of M3 of CheA at 303 K (left panel) and 283 K (right panel), in the presence of various concentrations of CheY, simulated using the transition rates in Fig. 4a,b, with the same colors as in (**a**).

The resonances from M3 shifted non-linearly upon the addition of CheY (Fig. 5a). The ^1^H chemical shift perturbations observed for M3, as well as I5, upon the addition of a stoichiometric amount of CheY, which predominantly reflect the P1-CheY bound state, at 283 K were smaller than those at 303 K (Fig. 5a). For the further evaluation of the equilibrium constants, which were estimated using the resonances from I5, we performed simulations of the resonances from M3, as well as the resonances from other residues in the P1 domain of CheA, using the estimated equilibrium constants. As a result, the simulated signals were in good agreement with the experimentally observed resonances (Fig. 5b). From these results, we concluded that the equilibrium between the P1-CheY unbound state and the P1-CheY bound state shifted toward the former at a lower temperature.

### Binding mode of the P1-CheY bound state revealed by cross-saturation experiments

**Figure 6:**
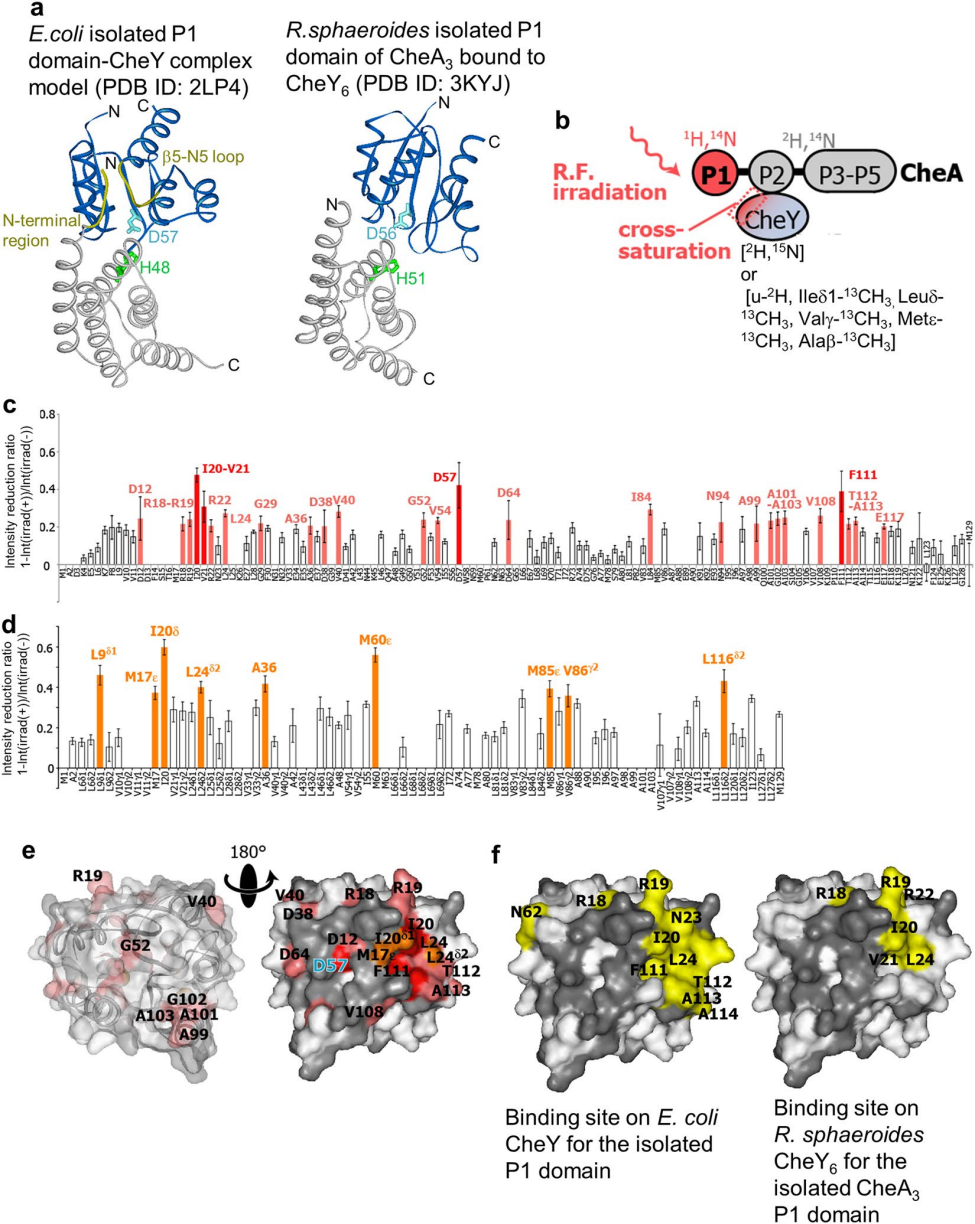
P1 domain-binding interface on CheY revealed by cross-saturation experiments utilizing segmentally labeled CheA. (**a**) Model of the *E. coli* isolated P1 domain-CheY complex (PDB ID: 2LP4) and the crystal structure of the *R. sphaeroides* isolated P1 domain of CheA_3_ bound to CheY_6_ (PDB ID: 3KYJ). The N-terminal region (Met1-Ser6) of the *E. coli* isolated P1 domain and the β5-α5 loop of CheY (Val108-Ala113) are yellow. (**b**) Schematic diagram of the experiments. (**c**) Plots of the reduction ratios of the signal intensities originating from the amide groups, with and without presaturation, in the cross-saturation experiments using [^1^H]-P1/[^2^H]-P2–5 CheA and [^2^H, ^15^N] CheY. Red and light orange plots represent the residues with signal intensity reduction ratios > 0.3 and within the 0.2–0.3 range, respectively. (**d**) Plots of the reduction ratios of the signal intensities originating from the methyl groups, with and without presaturation, in the cross-saturation experiments using [^1^H]-P1/[^2^H]-P2–5 CheA and [u-^2^H, Ileδ1-^13^CH_3_, Leuδ-^13^CH_3_, Valγ-^13^CH_3_, Metε-^13^CH_3_, Alaβ-^13^CH_3_] CheY. Orange plots represent the residues with signal intensity ratios > 0.35, and are labeled. In (**c**) and (**d**), the residues with reduction ratios < 0.2 and < 0.35, respectively, are white, and the error bars represent the root sum square of the reciprocal of the signal-to-noise ratio of the resonances with and without irradiation. (**e**) Mapping of the residues on CheY affected by the irradiation in the cross-saturation experiments (PDB ID: 1EAY). The V21, R22, G29, A36, V54, I84, N94, and L116 residues are hidden in these views. The residues with amide proton signal intensity reduction ratios > 0.3 and within the 0.2–0.3 range are colored red and light orange, respectively, and the residues with methyl proton signal intensity reduction ratios > 0.35 are colored orange. Proline residues and the residues with intensity reductions that were not determined, because of low signal intensity or spectral overlap, are gray. In the left view, the surface of CheY is transparent, and the ribbon diagrams are simultaneously displayed. (**f**) Isolated P1 domain-binding interfaces in the model of the *E. coli* isolated P1 domain-CheY (PDB ID: 2LP4) and the crystal structure of the *R. sphaeroides* isolated P1 domain of CheA_3_ bound to CheY_6_ (PDB ID: 3KYJ), mapped on the structure of CheY (PDB ID: 1EAY). Residues with (Σ*r*_*i*_^−6^)^−1/6^ < 5 Å, where *r*_*i*_ is the distance between each observed atom and the *i*th proton of the binding partner, are yellow. The residues with intensity reductions that were not determined in the cross-saturation experiments are gray. The molecular diagrams were generated with Web Lab Viewer Pro (Molecular Simulations, Inc.).

Mo *et al*. proposed a model of the complex composed of the isolated P1 and P2 domains and CheY from *E. coli*, based on their NMR study^33^. In this model, the relative orientation of the P1 domain and CheY was different from that in the crystal structures of its ortholog, the *Rhodobacter sphaeroides* CheA_3_P1-CheY_6_ complex^32^, and its structural homologue, the yeast Ypd1-Sln1(R1) complex^48^. The relative orientation of the *E. coli* isolated P1-isolated P2-CheY complex model allows the interaction between the N-terminal region of the P1 domain and the β5-α5 loop of CheY, whereas the corresponding residues are not present in the binding interface of the *R. sphaeroides* CheA_3_P1-CheY_6_ complex (Fig. 6a). It is possible that the binding mode difference is due to the absence of the linker connecting the P1 and P2 domains of CheA. Therefore, we performed cross-saturation experiments^49–53^ to identify the residues in the binding interface of the P1-CheY bound state. The experiments were performed under the conditions where > 80% of the observed CheA or CheY forms a 1:1 complex, as judged from the aforementioned titration experiments.

The binding interface on CheY for the P1 domain of CheA was determined by the cross-saturation experiments of either [^2^H, ^15^N] CheY or [u-^2^H, Ileδ1-^13^CH_3_, Leuδ-^13^CH_3_, Valγ-^13^CH_3_, Metε-^13^CH_3_, Alaβ-^13^CH_3_] CheY in complex with [^1^H]-P1/[^2^H]-P2–5 CheA (Fig. 6b). The amide groups of D12, R18-R22, L24, G29, A36, D38, V40, G52, V54, D57, D64, I84, N94, A99, A101-A103, V108, F111-A113, and E117 (Fig. 6c) and the methyl groups of L9, M17, I20, L24, A36, M60, M85, V86, and L116 were affected by irradiation (Fig. 6d), and the affected residues formed a continuous surface on the region surrounding D57 of CheY (Fig. 6e).

**Figure 7:**
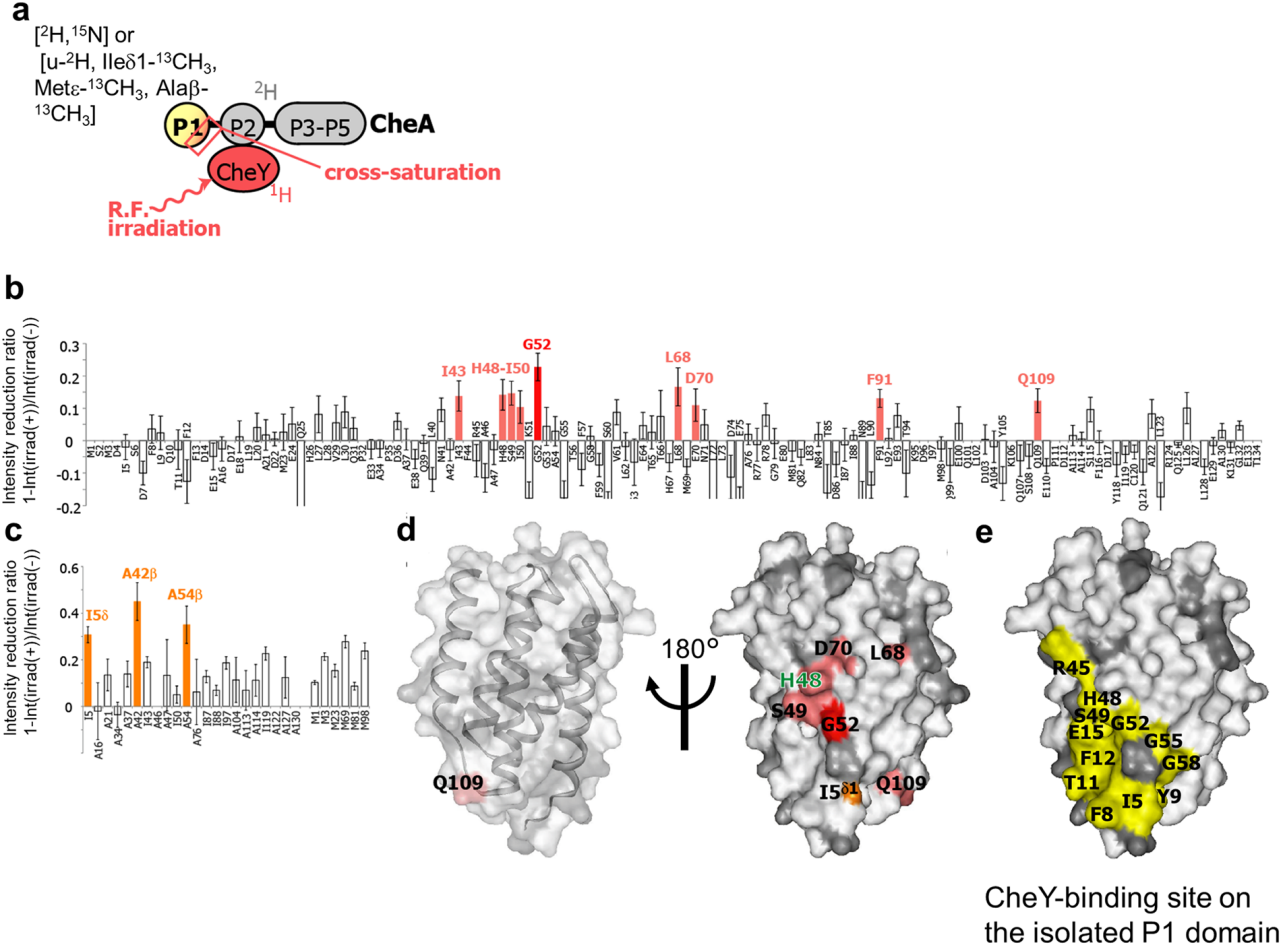
CheY-binding interface on the P1 domain of CheA revealed by cross-saturation experiments using segmentally labeled CheA. (**a**) Schematic diagrams of the experiments. (**b**) Plots of the reduction ratios of the signal intensities originating from the amide groups. Red and light orange plots represent the residues with signal intensity reduction ratios > 0.2 and within the 0.1–0.2 range, respectively, and are labeled. (**c**) Plots of the reduction ratios of the signal intensities originating from the methyl groups in the cross-saturation experiments using [u-^2^H, Ileδ1-^13^CH_3_, Metε-^13^CH_3_, Alaβ-^13^CH_3_] CheA and non-labeled CheY. Orange plots represent the residues with signal intensity reduction ratios > 0.3. In (**b**,**c**), the residues with reduction ratios < 0.1 and < 0.3 are white, respectively, and the error bars represent the root sum square of the reciprocal of the signal-to-noise ratio of the resonances with and without irradiation. (**d**) Mapping of the residues on the P1 domain of CheA affected by the irradiation in the cross-saturation experiments (PDB ID: 1I5N). The residues with amide proton signal intensity reduction ratios > 0.2 and within the 0.1–0.2 range are colored red and light orange, respectively, and the residues with methyl proton signal intensity reduction ratios > 0.3 are colored orange. Proline residues and residues with intensity reductions that were not determined, because of low signal intensity or spectral overlap, are gray. In the left view, the surface of the P1 domain is transparent, and the ribbon diagram is simultaneously displayed. F91, A42β, and A54β, which are close to H48, are hidden in these views. (**e**) CheY-binding interface in the model of the *E. coli* isolated P1 domain-CheY (PDB ID: 2LP4), mapped on the structure of the P1 domain (PDB ID: 1I5N). The labeling and coloring schemes are the same as in Fig. 6f. The molecular diagrams were generated with Web Lab Viewer Pro (Molecular Simulations, Inc.).

The CheY-binding interface on the P1 domain of CheA was determined by the cross-saturation experiments of either [^2^H, ^15^N]-P1/P2–5 CheA or [u-^2^H, Ileδ1-^13^CH_3_, Metε-^13^CH_3_, Alaβ-^13^CH_3_]-P1/P2–5 CheA in complex with non-labeled CheY (Fig. 7a). The residues significantly affected by irradiation were the amide groups of I43, H48-I50, G52, L68, D70, F91, and Q109 (Fig. 7b) and the methyl groups of I5, A42, and A54 (Fig. 7c). The affected residues formed a continuous surface on the region surrounding H48 of the P1 domain of CheA, and are mapped in Fig. 7d.

The I5 residue of CheA and the residues in the α5-β5 loop of CheY, V108 and F111-A113, which are not involved in the binding interface of the *R. sphaeroides* CheA_3_P1-CheY_6_ complex, were included in the binding interface of the CheA-CheY complex, although most of the residues in the N-terminal region of the P1 domain were not observed, probably due to fast exchange with solvent water (Figs 6–7). These results suggested that the relative orientation of the P1 domain of CheA and CheY is similar to that of the *E. coli* isolated P1 domain-isolated P2 domain-CheY complex model. We also confirmed that the binding mode of the P2 domain of CheA and CheY is similar to that of the isolated P2 domain-CheY complex, by cross-saturation experiments in which the binding interfaces on both of the P1 and P2 domains of CheA for CheY were observed (Supplementary Note and Figs S7–8).

## Discussion

Our NMR experiments revealed that CheA and CheY exist in equilibrium between the free state, the P1-CheY unbound state, the P1-CheY bound state, and the doubly-bound state (Fig. 1). This equilibrium would not be observed in experiments using the isolated P1 and P2 domains, because the linker connecting the P1 and P2 domains is required for this equilibrium.

**Figure 8:**
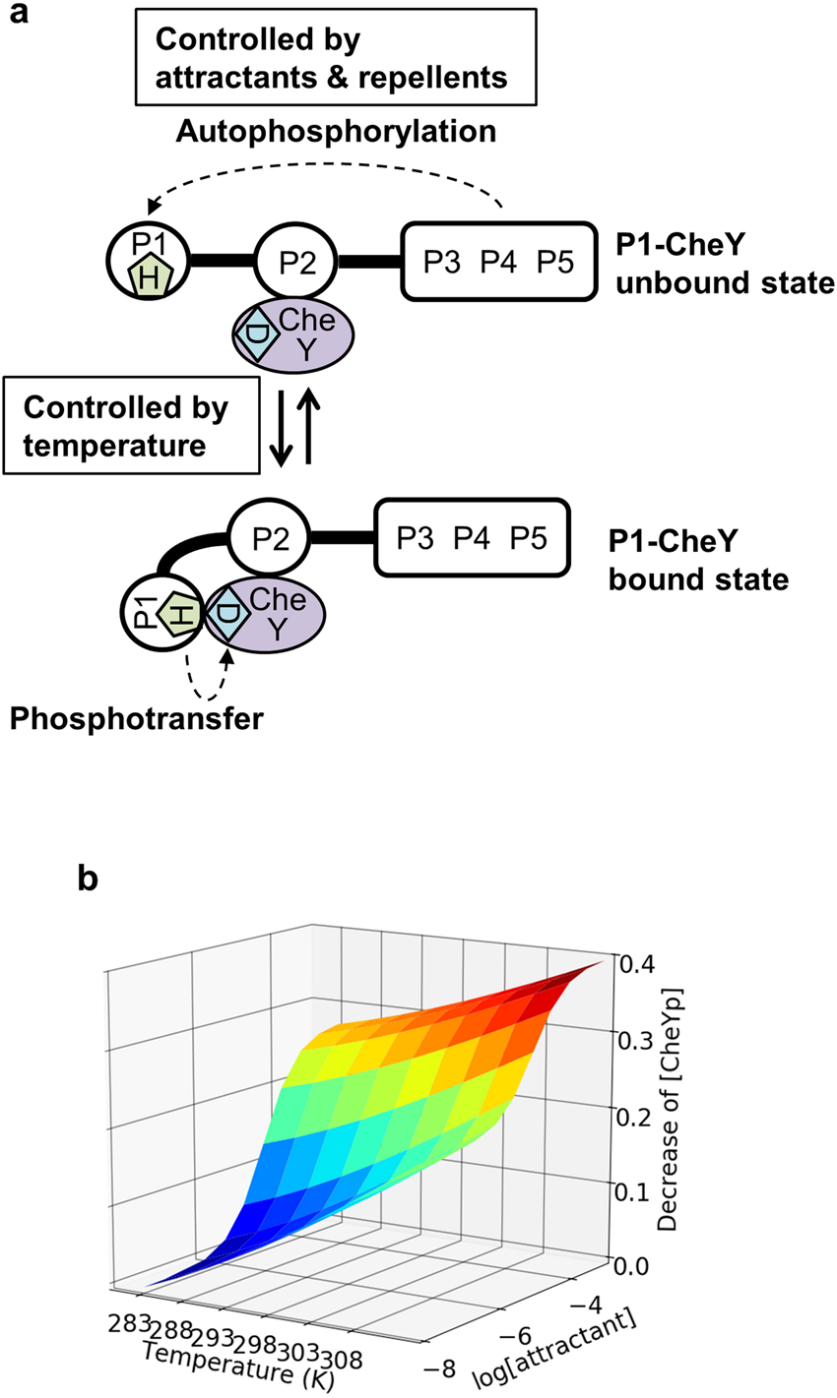
Dynamic domain arrangement of the CheA-CheY complex and its effects on chemotaxis signaling. (**a**) Schematic diagrams of the dynamic domain arrangement of the CheA-CheY complex at physiological concentrations of CheA and CheY. (**b**) 3D contour plot of the calculated decrease in the amount of phosphorylated CheY upon increasing the attractant concentration and/or temperature.

In the crystal structure of the *R. sphaeroides* CheA_3_P1-CheY_6_ complex, the distance between the residues corresponding to H48 of *E. coli* CheA and D57 of *E. coli* CheY is 7.5 Å^32^, whereas it should be less than 4.9 Å for the phosphotransfer reactions to proceed by an associative mechanism^54^. Our cross-saturation experiments revealed that the N-terminal region of the P1 domain of CheA and the β5-α5 loop of CheY, which are not involved in the binding interface in the crystal structure of the *R. sphaeroides* CheA_3_P1-CheY_6_ complex, are included in the binding interface of the P1-CheY bound state (Figs 6,7). In addition, both H48 of CheA and D57 of CheY participate in the binding interface (Figs 6,7). These results suggested that, in the P1-CheY bound state, H48 of CheA and D57 of CheY are sufficiently close to each other to effectively allow the phosphotransfer reaction to proceed from H48 of CheA to D57 of CheY (Fig. 8a). In contrast, the autophosphorylation of the P1 domain by the P4 domain, which occurs in the P1-CheY unbound state, does not occur in the P1-CheY bound state, due to steric hindrance (Fig. 8a). Therefore, the equilibrium between the P1-CheY unbound state and the P1-CheY bound state, with fast exchange rates, would be important for both the autophosphorylation and phosphotransfer reactions with the P4 domain and CheY, respectively. This dynamic domain arrangement of the CheA-CheY complex simultaneously affects these reaction rates. The importance of the dynamic domain arrangement of the CheA-CheY complex for the signaling is exemplified by the *in vitro* signaling assay: the amount of phosphorylated CheY was decreased by the I20A mutation of CheY, which disrupts the P1 domain-CheY interaction of the CheA-CheY complex (Fig. 1h,i).

The equilibrium between the P1-CheY unbound state and the P1-CheY bound state exhibited the temperature-dependent population shift (Figs 2–4), and thus it is possible that the temperature-dependent population shift of the dynamic domain arrangement of the CheA-CheY complex regulates the concentration of phosphorylated CheY. Our quantitative time-course simulation of the signaling, based on the equilibrium constants at 283 K and 303 K, revealed that the temperature-dependent population shift of the dynamic domain arrangement of the CheA-CheY complex induces a ~30% change in the concentration of phosphorylated CheY, which is comparable to the changes induced by attractants or repellents (Fig. 8b and Supplementary Table S2 ^55^). In addition, the enthalpy change of the equilibrium between the P1-CheY unbound state and the P1-CheY bound state, calculated using the equilibrium constants at 283 K and 303 K and the van’t Hoff equation, was ~30 kJ/mol (10 ~ 40 kJ/mol), which is comparable to the previously reported overall activation energy of the CheY phosphorylation^24^. These results suggested that the dynamic domain arrangement of the CheA-CheY complex functions as the primary temperature sensor for bacterial thermotaxis. The above-described calculation indicated that the concentration of phosphorylated CheY decreases with an increase in the temperature, due to the inhibition of the autophosphorylation. This is in agreement with the warm-seeking thermotaxis at < 310 K^3^.

Cells that express only one of the chemoreceptors (Tsr, Tar, Trg, and Tap) reportedly exhibited different thermal responses, which are affected by both the ligand binding and methylation of the chemoreceptors^1,3–8^. These effects of the modifications of the chemoreceptors on the thermotaxis can be explained by the temperature-dependent shift of the dynamic domain arrangement of the CheA-CheY complex (Supplementary Note and Supplementary Fig. S9).

The direction of cell movement reportedly switches between heat-seeking and cold-seeking at ~310 K, for accumulation at the preferred temperature^1^. At temperatures above 303 K, the temperature-dependent modulation of the conformation of chemoreceptor trimers was observed, in the previously reported FRET analyses of the chemoreceptors with a YFP tag^23^. Therefore, we propose that the dynamic domain arrangement of the CheA-CheY complex regulates the warm-seeking behavior at temperatures below the critical temperature, and the conformation of the chemoreceptor trimer regulates the cold-seeking behavior at temperatures above the critical temperature.

We can imagine that the temperature also regulates the autophosphorylation reaction rate of CheA, in which the P1 domain binds to the P4 domain. However, we could not observe the interaction between the P4 and P1 domains by either the cross-saturation or paramagnetic relaxation enhancement experiments of full-length CheA, suggesting that the population with interacting P1 and P4 domains is less than 10%. This is in agreement with the slow autophosphorylation rates (~ 1 min^−1^) of CheA in the absence of CheW and chemoreceptors. The population of the P1-P4 bound state of full-length CheA would be increased upon complexation with CheW and chemoreceptors^11,56–61^, and structural analyses of the ternary complex would provide information about the mechanism underlying the regulation of the autophosphorylation rates.

Other two-component systems, such as the DesK-DesR signal transduction system, also reportedly sense the environmental temperature^62^. Whereas the transmembrane region of DesK, which corresponds to the chemoreceptor of the CheA-CheY signaling system, is proposed to sense the temperature-dependent change of the bilayer thickness^63,64^, it is possible that other two-component system proteins sense the temperature by the temperature-dependent population shift of the dynamic domain arrangement of the histidine kinase-response regulator complexes, similar to the CheA-CheY complex.

The dynamic domain arrangement of the full-length CheA-CheY complex, which enables the multi-step signaling reactions and the temperature-dependent regulation of signaling, originates from the weak interaction between the P1 domain of CheA and CheY. Although little is known about weak protein-protein interactions with association constants lower than 10^4^ M^−1^, due to the lack of appropriate methods to detect and characterize such interactions, NMR analyses of the dynamic domain arrangements within multidomain protein complexes would be useful for understanding their functions.

## Materials and Methods

### Sample preparation

The *chea*, *chey*, *chew*, and *tar* genes were amplified from the genomic DNA of *E. coli* strain BL21 by PCR reactions, and transferred into the pET43a vector (Novagen) using the *Nde*I and *Hind*III restriction sites. Mutagenesis of *chea* and *chey* was performed using a QuikChange site-directed mutagenesis kit (Stratagene). Plasmids for the expression of the isolated P1 domain were constructed by mutating the codon for P135 of CheA (CCA) to the stop codon (TGA). For the construction of the plasmid for the expression of the isolated P2 domain, the gene encoding the P2 domain of CheA (residues S156 to S229) was amplified by PCR reactions and transformed into the pET28a vector, using the *Nde*I and *Xho*I restriction sites. A hexahistidine tag (CACCACCACCACCACCAC) was inserted at the 3′ end of the *tar* gene, using a QuikChange site-directed mutagenesis kit. pYMRSF01 and pYMRSF39, which were utilized for the preparation of the segmentally labeled CheA, were constructed as described previously^35^.

For the preparation of Tar and the segmentally labeled CheA, the *E. coli* strains RP3098 and ER2566 were utilized as the host strains, respectively. For the preparation of the other proteins, BL21(DE3) was utilized as the host strain. The cells were grown to mid-exponential phase at 37 °C. Non-labeled proteins were prepared by growing cells in LB medium. The [^13^C, ^15^N] CheY and the [^13^C, ^15^N] isolated P1 domain were prepared by growing cells in M9 medium containing ^15^NH_4_Cl (1 g/liter) and glucose-^13^C_6_ (2 g/liter), supplemented with Celtone-CN powder (1 g/liter). The [^2^H, ^15^N] CheA and [^2^H, ^15^N] CheY were prepared using M9 medium in 99.9% deuterium oxide containing ^15^NH_4_Cl (1 g/liter) and glucose-d_7_ (2 g/liter), supplemented with Celtone-DN powder (1 g/liter). The [^2^H, ^13^C, ^15^N] CheA was prepared by growing cells in M9 medium in 99.9% deuterium oxide containing ^15^NH_4_Cl (1 g/liter) and glucose-^13^C_6_-d_7_ (2 g/liter), supplemented with Celtone-DCN powder (1 g/liter). The [u-^2^H, Ileδ1-^13^CH_3_, Leuδ-^13^CH_3_, Valγ-^13^CH_3_] CheA and CheY were prepared by growing cells in M9 medium in 99.9% deuterium oxide containing [u-^2^H] glucose (2 g/liter), and with 50 mg/L [4-^13^C, 3, 3-d_2_]-α-ketobutyrate (CIL) and 120 mg/L [dimethyl-^13^C_2_]-α-ketoisovalerate (CIL), added 30 min prior to induction. The [u-^2^H, Ileδ1-^13^CH_3_, Metε-^13^CH_3_, Alaβ-^13^CH_3_] CheY was prepared by growing cells in M9 medium in 99.9% deuterium oxide containing 200 mg/L [2-^2^H, 3-^13^C]-alanine, 100 mg/L [α,β,β -^2^H_3_, methyl-^13^C]-methionine, 2.5 g/L succinate-d_4_ (CIL), and 60 mg/L [4-^13^C, 3, 3-d_2_]-α-ketobutyrate, added 30 min prior to induction. The [α, β, β-^2^H_3_, methyl-^13^C]-methionine and [α-^2^H, methyl-^13^C]-alanine were synthesized by the enzymatic deuteration of [methyl-^13^C]-alanine (ISOTEC) and [methyl-^13^C]-methionine (CIL), with *Escherichia coli* tryptophan synthase and cystathionine-γ-synthase, respectively, as previously described^65,66^. The [Leu/Val-^13^CH_3_^pro-S^] CheY was prepared by growing cells in M9 medium in 99.9% deuterium oxide containing 300 mg/L [2-^13^C methyl-4-^2^H_3_] acetolactate (NMR-BIO), added 45 min prior to induction as previously described^67^.

The protein expression was induced by the addition of isopropyl β-D-1-thiogalactopyranoside to a final concentration of 0.5 mM. After the induction at 25 °C for 6 hours, the cells were harvested and re-suspended in buffer C (20 mM sodium phosphate, pH 8.0, 300 mM NaCl). The full-length CheA and CheY and the isolated P1 and P2 domains were purified, and the membrane fraction containing Tar was prepared as described previously^68^. The [^2^H, ^15^N]P1/P2–5 CheA, [u-^2^H, Ileδ1-^13^CH_3_, Metε-^13^CH_3_, Alaβ-^13^CH_3_]-P1/[^2^H]-P2–5 CheA, and [u-^2^H, Ileδ1-^13^CH_3_, Metε-^13^CH_3_, Alaβ-^13^CH_3_]-P1/[^2^H]-P2–5 CheA were prepared as described previously^35^. Protein concentrations are expressed as monomer concentrations.

### Phos-tag SDS-PAGE Assay

Twenty picomoles of the wild-type CheA, CheW, and the membrane fraction containing Tar were dissolved in 8 μL of buffer E (50 mM Tris (pH 9.0), 5 mM MgCl_2_, 50 mM KCl, and 0.5 mM dithiothreitol (DTT)). The reaction mixtures were incubated at room temperature for more than 3 hours, and then 1 μL of 200 μM wild-type or the I20A mutant of CheY was added to the reaction mixture. In the negative control experiment, L-aspartic acid, which binds to Tar and inhibits the phosphorylation of CheY, was also added to a final concentration of 0.5 mM. The reaction mixtures were further incubated for 10 minutes, and the autophosphorylation was initiated by the addition of 1 μL of 30 mM ATP. After a 10 second incubation, the reaction was terminated by the addition of an equivalent volume of 2 × SDS electrophoresis sample buffer (125 mM Tris, pH 6.8, 4% SDS, 20% glycerol, and 0.001% bromophenol blue). For the preparation of phosphorylated CheY, 20 μM (final concentration) CheY was dissolved in buffer E with 10 mM acetyl phosphate (Sigma). The reaction mixtures were incubated at room temperature for 7 min, followed by the addition of an equivalent volume of 2 × SDS electrophoresis sample buffer to stop the reaction. These samples were subjected to 12% Zn^2+^- or 18% Mn^2+^-phos-tag SDS-PAGE analysis with 50 μM Phos-tag^69^. The bands corresponding to CheY were quantified using NIH ImageJ software.

### ITC Analyses

ITC experiments were performed, using a MicroCal iTC200 calorimeter (GE Healthcare), with stirring at 1,000 rpm at 30 °C. The protein samples were dialyzed against a buffer containing 20 mM sodium phosphate, pH 7.5, and 5 mM MgCl_2_. The titration of the CheA with CheY involved 19 injections of 2 μl of the CheY solution (0.46–1.1 mM) at intervals of 90 or 120 s into a sample cell containing 200 μl of CheA (50–90 μM). The heat of dilution of the titrant (CheY) was subtracted from the titration data for the CheY titration into CheA. The data were analyzed with the MicroCal Origin^TM^ 5.0 software to determine the stoichiometry (N), dissociation constants (K_d_), enthalpy changes (ΔH), and entropy changes (ΔS) by fitting a single site binding model to the thermal titration data.

### NMR spectroscopy

Sequential assignments of the backbone resonances of the P1 and P2 domains in full-length CheA were achieved by TROSY-HNCA, TROSY-HN(CO)CA, intra-HNCA, TROSY-HNCACB, and TROSY-HN(CO)CACB experiments^70–74^, performed at 303 K on a Bruker Avance 800 spectrometer equipped with a cryogenic probe. For the analyses, 0.3 mM [^2^H, ^13^C, ^15^N] CheA was dissolved in 50 mM Tris, pH 7.5, in H_2_O/D_2_O = 90/10. Sequential assignments of the backbone resonances of CheY were achieved by HNCACB and HN(CO)CACB, performed at 303 K on a Bruker Avance 500 spectrometer equipped with a cryogenic probe. For the analyses, 2.0 mM [^13^C, ^15^N] CheY was dissolved in 50 mM Tris, pH 6.7, and 5 mM MgCl_2_, in H_2_O/D_2_O = 90/10. Assignments of the resonances from the methyl groups of the isolated P1 domain and CheY were achieved by 3D (H)CCH-TOCSY, HMBC^75^, LRCH, LRCC, and HBHA(CO)NH, performed at 303 K on a Bruker Avance 500 or Avance 600 spectrometer equipped with a cryogenic probe. For the analyses, 0.78 mM [^13^C, ^15^N] isolated P1 domain and 0.3 mM [^13^C, ^15^N] CheY were dissolved in 20 mM sodium phosphate, pH 7.1, and 2 mM DTT, in H_2_O/D_2_O = 90/10. Stereospecific assignments of the methyl groups of the leucine and valine residues of CheY were achieved using the spectra of 0.2 mM [Leu/Val-^13^CH_3_^pro-S^] CheY, dissolved in 20 mM sodium phosphate, pH 6.6, and 2 mM DTT, in H_2_O/D_2_O = 1/99. Assignments of the methyl groups of the methionine residues of CheY were reported previously^76^. All spectra were processed by Topspin, version 2.1, and analyzed by SPARKY (T.D. Goddard and D.G. Kneller, University of California, San Francisco) or CARA (Rochus Keller).

The ^1^H-^15^N heteronuclear single quantum coherence (HSQC) or TROSY spectra of 0.1 mM [^2^H, ^15^N] isolated P1, [^2^H, ^15^N] isolated P2, and [^2^H, ^15^N] CheA, combined with the non-labeled wild type or I20A mutant of CheY, were recorded on a Bruker Avance 500 spectrometer equipped with a cryogenic probe at 303 K. The samples were dissolved in 50 mM Tris, pH 7.5, in H_2_O/D_2_O = 90/10.

The cross-saturation experiments were performed on a Bruker Avance 600 or 800 spectrometer equipped with a cryogenic probe. In the amide-directed cross-saturation experiments for the identification of the residues of CheA in close proximity to CheY, 0.1 mM non-labeled CheY was combined with 0.1 mM [^2^H, ^15^N] CheA or [^2^H, ^15^N]-P1/P2–5 CheA in 50 mM Tris, pH 6.5, in H_2_O/D_2_O = 20/80. In the methyl-directed cross-saturation experiments for the identification of the residues of CheA in close proximity to CheY, 0.05 mM non-labeled CheY was combined with 0.05 mM [u-^2^H, Ileδ1-^13^CH_3_, Metε-^13^CH_3_, Alaβ-^13^CH_3_]-P1/[^2^H]-P2–5 in 20 mM sodium phosphate, pH 7.8, 5 mM MgCl_2_, and 2 mM DTT, in H_2_O/D_2_O = 1/99. In the amide-directed cross-saturation experiments for the identification of the residues of CheY in close proximity to CheA, 0.2 mM non-labeled CheA or P1/[^2^H]-P2–5 CheA was combined with 0.15 mM [^2^H, ^15^N] CheY. The former and the latter samples were dissolved in 50 mM Tris, pH 6.6, 5 mM MgCl_2_, 2 mM DTT, 150 mM KCl, 5 mM AMP-PNP, and 1 mM sodium 3-(trimethylsilyl)-1-propanesulfonate, in H_2_O/D_2_O = 20/80. In the methyl-directed cross-saturation experiments for the identification of the residues of CheY in close proximity to CheA, 0.23 mM P1/[^2^H]-P2–5 CheA was combined with 0.15 mM [u-^2^H, Ileδ1-^13^CH_3_, Metε-^13^CH_3_, Alaβ-^13^CH_3_] CheY in 20 mM NaPi, pH 7.0, 5 mM MgCl_2_, 150 mM KCl, and 2 mM DTT, in H_2_O/D_2_O = 1/99. The amide-directed cross-saturation experiments were performed using the reported pulse scheme^51^, with a minor modification from the 3-9-19 selective pulse to soft-90 hard-180 soft-90 pulse elements. The saturation for the aliphatic protons of CheY was accomplished using the WURST-2 decoupling scheme. The saturation frequency was set at 1.5 ppm, and the maximum radiofrequency amplitude was 0.17 kHz for WURST-2 (adiabatic factor Q_0_ = 1). The saturation times were set to 0.5 s. The total relaxation delay (saturation time + relaxation delay) was set to 6.0 s. The methyl-directed cross-saturation experiments were performed using the reported pulse scheme^77^. In the methyl-directed cross-saturation experiments, the irradiation frequency was set to 6.9 ppm, and the maximum radiofrequency amplitude was 0.21 kHz for WURST-20 (adiabatic factor Q_0_ = 1). The saturation times were set to 1.25 s. The total relaxation delay (saturation time + relaxation delay) was set to 3.0 s.

### *In situ* chemical shift error analysis

Synthetic 2D time domain data, composed of 100 synthetic signals with relaxation rates and S/N ratios similar to those of the observed resonances, were generated using in-house developed programs. The data were processed in the same manner as the observed data, and the standard deviations of the chemical shifts were calculated.

### Simulations

The simulations were performed by in-house developed programs, written in the Python 2.7 programming language, supplemented with the extension modules Numpy 1.8, Scipy 0.14, and Cython 0.20. The programs were run on 16 Intel Xenon X5687 3.60 GHz CPUs operating under CentOS 5.8.

The 2D simulated NMR data were generated via the analytical solutions of the McConnel equations^78^, as reported previously^79,80^. The association rate constants were set to 2 × 10^8^ M^−1^s^−1^, 10,000 s^−1^, and 10^7^ M^−1^s^−1^, respectively, which are sufficiently large to exhibit fast exchange regimes. The ^1^H and ^13^C transverse relaxation rates at 303 K in the free state were set to 45 s^−1^ and 12 s^−1^, respectively, and those in the bound states were set to 60 s^−1^ and 15 s^−1^. At 283 K, the ^1^H and ^13^C transverse relaxation rates in the free state were set to 70 s^−1^ and 40 s^−1^, respectively, and those in the bound states were set to 100 s^−1^ and 60 s^−1^. The aforementioned association rate constants and relaxation rates were treated as constants in the following analyses.

The dissociation constants, the ^1^H and ^13^C chemical shifts of the P1-CheY bound state and the doubly bound states, and their errors at 283 K and 303 K were calculated using the following Markov-chain Monte-Carlo algorithm. Firstly, the simulated 2D spectra under *k* conditions with various amounts of CheY, *A*_*k*_ (*v*), were generated using arbitrary initial parameters. Secondly, the spectra were fit to the following 2D Lorentzian peak:

1 $${A}_{k}({\nu }_{1H},{\nu }_{13C})={\rm{I}}\cdot \frac{{T}_{2,13C}}{1+2\pi {({\nu }_{13C}-{\nu }_{k,13C})}^{2}{{T}_{2,13C}}^{2}}\cdot \frac{{T}_{2,1H}}{1+2\pi {({\nu }_{1H}-{\nu }_{k,1H})}^{2}{{T}_{2,1H}}^{2}}\,$$

where I is the intensity and T_2,13C_, T_2,1H_, *v*_k,13C_ and m *v*_k,1H_ are the ^1^H and ^13^C relaxation times and the frequencies of the peak. Thirdly, the posterior probability *P* was calculated by the following equations:

2 $${\rm{P}}(0)=\prod _{k}\frac{1}{\sigma \sqrt{2\pi }}{e}^{\frac{{({v}_{k,13C}-{v}_{k,13C,obs})}^{2}}{2{\sigma }^{2}}}\prod _{k}\frac{1}{\sigma \sqrt{2\pi }}{e}^{\frac{{({v}_{k,1H}-{v}_{k,1H,obs})}^{2}}{2{\sigma }^{2}}}$$

where *v*_*k*,1*H*_,_*obs*_ and *v*_*k*,13*C*_,_*obs*_ are the ^1^H and ^13^C frequencies of the experimentally observed signals, respectively. σ was set to ~1 Hz. Fourthly, one of the above-described parameters was randomly changed. The maximum ^1^H and ^13^C chemical shift differences between 283 K and 303 K were set to 0.01 and 0.05 ppm, respectively. Fifthly, the proposed parameter was accepted with the following probabilities:

3 $$min(1,\frac{P(1)}{P(0)})$$

The calculation was performed for all parameters, and repeated 20,000 times. The average and the standard deviation of the parameters in the last 10,000 calculations were utilized as the estimated values and their errors, respectively.

In the quantitative calculation of the intracellular signaling, a differential algebraic model of the bacterial chemotaxis signaling was formulated according to the literature^81–84^, with modification of the reaction rates of the autophosphorylation and phosphotransfer. The CheA-CheY phosphotransfer reaction rate was set to be proportional to the population of the P1-CheY unbound state, p_b_, and the autophosphorylation and CheA-CheB phosphotransfer reaction rates were set to be proportional to the population of the P1-CheY unbound state, (1- p_b_), considering the fact that the formation of the catalytic complex is the rate-limiting step of these reactions^13,84^.

4 $${\alpha }_{0}=\frac{{p}_{0}^{L}[L]}{{K}_{L}+[L]}+\frac{{p}_{0}{K}_{L}}{{K}_{L}+[L]}$$

5 $${\alpha }_{1}=\frac{{p}_{1}^{L}[L]}{{K}_{L}+[L]}+\frac{{p}_{1}{K}_{L}}{{K}_{L}+[L]}$$

6 $${\alpha }_{2}=\frac{{p}_{2}^{L}[L]}{{K}_{L}+[L]}+\frac{{p}_{2}{K}_{L}}{{K}_{L}+[L]}$$

7 $${\alpha }_{3}=\frac{{p}_{3}^{L}[L]}{{K}_{L}+[L]}+\frac{{p}_{3}{K}_{L}}{{K}_{L}+[L]}$$

8 $${\alpha }_{4}=\frac{{p}_{4}^{L}[L]}{{K}_{L}+[L]}+\frac{{p}_{4}{K}_{L}}{{K}_{L}+[L]}$$

9 $$\frac{d[{T}_{0}]}{dt}=-(1-{\alpha }_{0})\frac{{k}_{r}[R]}{{K}_{R}+{T}^{I}}[{T}_{0}]+{\alpha }_{1}\frac{{k}_{b}[{B}_{p}]}{{K}_{B}+{T}^{A}}[{T}_{1}]$$

10 $$\frac{d[{T}_{1}]}{dt}=-(1-{\alpha }_{1})\frac{{k}_{r}[R]}{{K}_{R}+{T}^{I}}[{T}_{1}]+{\alpha }_{2}\frac{{k}_{b}[{B}_{p}]}{{K}_{B}+{T}^{A}}[{T}_{2}]+(1-{\alpha }_{0})\frac{{k}_{r}[R]}{{K}_{R}+{T}^{I}}[{T}_{0}]-{\alpha }_{1}\frac{{k}_{b}[{B}_{p}]}{{K}_{B}+{T}^{A}}[{T}_{1}]$$

11 $$\frac{d[{T}_{2}]}{dt}=-(1-{\alpha }_{2})\frac{{k}_{r}[R]}{{K}_{R}+{T}^{I}}[{T}_{2}]+{\alpha }_{3}\frac{{k}_{b}[{B}_{p}]}{{K}_{B}+{T}^{A}}[{T}_{3}]+(1-{\alpha }_{1})\frac{{k}_{r}[R]}{{K}_{R}+{T}^{I}}[{T}_{1}]-{\alpha }_{2}\frac{{k}_{b}[{B}_{p}]}{{K}_{B}+{T}^{A}}[{T}_{2}]$$

12 $$\frac{d[{T}_{3}]}{dt}=-(1-{\alpha }_{3})\frac{{k}_{r}[R]}{{K}_{R}+{T}^{I}}[{T}_{3}]+{\alpha }_{4}\frac{{k}_{b}[{B}_{p}]}{{K}_{B}+{T}^{A}}[{T}_{4}]+(1-{\alpha }_{2})\frac{{k}_{r}[R]}{{K}_{R}+{T}^{I}}[{T}_{2}]-{\alpha }_{3}\frac{{k}_{b}[{B}_{p}]}{{K}_{B}+{T}^{A}}[{T}_{3}]$$

13 $$\frac{d[{T}_{4}]}{dt}=(1-{\alpha }_{3})\frac{{k}_{r}[R]}{{K}_{R}+{T}^{I}}[{T}_{3}]-{\alpha }_{4}\frac{{k}_{b}[{B}_{p}]}{{K}_{B}+{T}^{A}}[{T}_{4}]$$

14 $$\frac{d[{A}_{p}]}{dt}={k}_{auto}[{T}^{A}][A](1-{p}_{B})-{k}_{ApY}[{A}_{p}][Y]{p}_{B}-{k}_{ApB}[{A}_{p}][B](1-{p}_{B})$$

15 $$\frac{d[{Y}_{p}]}{dt}={k}_{ApY}[{A}_{p}][Y]{p}_{B}-{k}_{Yp}{Y}_{p}-{k}_{YpM}[M][{Y}_{p}]+{k}_{MYp}[M{Y}_{p}]-{k}_{YpZ}{Y}_{p}$$

16 $$\frac{d[M{Y}_{p}]}{dt}={k}_{YpM}[M][{Y}_{p}]-{k}_{MYp}[M{Y}_{p}]$$

17 $$\frac{d[{B}_{p}]}{dt}={k}_{ApB}[{A}_{p}][B](1-{p}_{B})-{k}_{Bp}[{B}_{p}]$$

18 $${[T]}^{A}={\alpha }_{0}[{T}_{0}]+{\alpha }_{1}[{T}_{1}]+{\alpha }_{2}[{T}_{2}]+{\alpha }_{3}[{T}_{3}]+{\alpha }_{4}[{T}_{4}]$$

19 $${[T]}^{I}={[T]}_{tot}-{[T]}^{A}$$

20 $$[A]={[A]}_{tot}-[{A}_{p}]$$

21 $$[Y]={[Y]}_{tot}-[{Y}_{p}]$$

22 $$[B]={[B]}_{tot}-[{B}_{p}]$$

23 $$[M]={[M]}_{tot}-[M{Y}_{p}]$$

The terms [*A*] and [*A*]_*p*_ denote the concentrations of unphosphorylated and phosphorylated CheA, respectively. [*Y*] and [*Y*]_*p*_ denote the concentrations of unphosphorylated and phosphorylated CheY, respectively. [*B*] and [*B*]_*p*_ denote the concentrations of unphosphorylated and phosphorylated CheB, respectively. [*M*] and [*MY*_*p*_] denote the concentrations of free FliM and the FliM-phosphorylated CheY complex, respectively. [*T*_0_], [*T*_1_], [*T*_2_], [*T*_3_], and [*T*_4_] denote the concentrations of chemoreceptors with zero, one, two, three, and four methylated residues, respectively. [*T*]^*A*^ and [*T*]^*I*^ denote the concentrations of the active and inactive chemoreceptors, respectively. The other parameters for the model are shown in Supplementary Table 2.

Numerical solutions of the differential algebraic equations were calculated with a time step of 10^−4^ s, using the Runge-Kutta method. The 10 s time-course at 283 K without the attractant was calculated, and the concentrations at 10 s were set to the initial concentrations in the calculations under the other conditions. The minimum phosphorylated CheY concentrations in the time-course, relative to the initial phosphorylated CheY concentration, were calculated as the decreases of the phosphorylated CheY.

### Data Deposition

Assigned chemical shifts for CheA and isolated P1 domain of CheA have been deposited in the Biological Magnetic Resonance Data Bank under accession code 27188 and 27189, respectively.

### Data Availability

The data that support the findings of this study are available from the corresponding author upon request.

## Electronic supplementary material

Supplementary Note and Figures
