# Supplementary material for: Dynamic domain arrangement of CheA-CheY complex regulates bacterial thermotaxis, as revealed by NMR
Source: Sci Rep. 2017 Nov 28;7:16462. doi: 10.1038/s41598-017-16755-x (PMC5705603; doi:10.1038/s41598-017-16755-x)
Supplement: Supplementary file 1 — Supplementary Note and Figures [file 41598_2017_16755_MOESM1_ESM.pdf]

**Supplementary File**

**"Dynamic domain arrangement of CheA-CheY complex regulates bacterial thermotaxis, as revealed by NMR"**

Yuichi Minato, Takumi Ueda, Asako Machiyama, Hideo Iwaï, and Ichio Shimada

## Supplementary Note

### Resonance assignments of the full-length CheA and CheA-CheY complex

NMR signal assignments for the main chain atoms of the P1 and P2 domains of the full-length CheA were accomplished, based on standard triple resonance experiments of [$^{2}$H, $^{13}$C, $^{15}$N] CheA. As a result, 95% (123/130) and 96% (64/67) of the backbone resonances from the P1 and P2 domains of CheA were assigned, respectively (Supplementary Fig. S2a-b). The chemical shifts of the resonances from the P1 and P2 domains of CheA were almost identical to those of the isolated P1 and P2 domains. Assignments for the methyl groups of 14 alanine, 7 isoleucine, and 6 methionine residues in the P1 domain of CheA were established by comparison with the spectra of the isolated P1 domain (Supplementary Fig. S2c-d). The assignments for the P1 and P2 domains of CheA and CheY in the CheA-CheY complex were accomplished by the titration experiments.

### Origin of the chemical shift perturbation observed for the P1 domain of CheA

Chemical shift perturbation was observed for the resonances from the P1 domain of CheA, upon the addition of a stoichiometric amount of CheY. In order to examine whether the perturbation is derived from the P1 domain-CheY interaction or the conformational

change induced by the P2 domain-CheY interaction, we created CheY mutants with decreased affinities for the P1 domain, and examined the CheY-induced chemical shift perturbation of the mutants. An alanine mutation was introduced into I20 of CheY, which is close to D57 and sufficiently distant from the P2 domain, in the previously reported structure of the isolated P2 domain-CheY complex[37], and the affinities of the CheY/I20A mutant for the full-length CheA and the isolated P2 domain were determined by isothermal titration calorimetry. As a result, the association constant of the CheY/I20A mutant for the full-length CheA was significantly lower than that of the wild type, whereas the affinity of the CheY/I20A mutant for the P2 domain was almost identical to that of the wild type (Supplementary Fig. S3a-d and Supplementary Table S1). Therefore, the P1 domain-CheY interaction was disrupted by introducing the I20A mutation in CheY.

NMR spectra of the [$^{2}$H, $^{15}$N]-P1/P2–5 CheA with a stoichiometric amount of the CheY/I20A were then recorded. As a result, the chemical shifts of the resonances from the P1 domain were almost identical to those in the absence of CheY (Supplementary Fig. S3e). In the $^{1}$H-$^{15}$N TROSY spectra of 100 μM [$^{2}$H, $^{15}$N]-P1/P2–5 CheA, in the presence of various concentrations of non-labeled CheY without the I20A mutation, the resonance from G55 exhibited a slight $^{1}$H downfield shift upon the addition of a stoichiometric

amount of CheY, and exhibited $^{1}$H and $^{15}$N downfield shifts upon the further addition of excess amounts of CheY (Supplementary Fig. S4a-c). The CheA-CheY interaction was also perturbed by introducing mutations of other residues of CheY, including N59K. Therefore, the CheY-induced chemical shift perturbation observed for the resonances from the P1 domain in the full-length CheA is derived from the P1 domain-CheY interaction.

**Interaction between the isolated P1 domain and CheY**

In the experiments using the isolated P1 domain (Supplementary Fig. S4d), instead of the full-length CheA, the resonances from G55 and other residues exhibited linear CheY-dependent shifts (Supplementary Fig. S4e-f), and the dissociation constant of the isolated P1 domain for CheY was calculated as 900 μM (700 ~ 1,100 μM) (Supplementary Fig. S4g).

**Binding mode of the P2 domain of CheA and CheY**

The binding interface on the P2 domain of CheA for CheY was determined by the cross-saturation experiments of [$^{2}$H, $^{15}$N] CheA and non-labeled CheY (Supplementary Fig. S7a). The E18, A21, I43, H48, K51, G53, G55, G58, A104, F116, L123, L175, L179,

L199, I208, A210, I216, A218, and I221 residues were affected by the irradiation (Supplementary Fig. S7b). Although we could not determine the intensity for many residues due to the signal overlaps, most of the affected residues, which are mapped in Supplementary Fig. S7c-d, were located at the CheY-binding site on the P1 domain of CheA (Fig. 7) and the CheY-binding site on the isolated P2 domain in the crystal structure of the isolated P2 domain-CheY complex (Supplementary Fig. S7e).

The binding interface on CheY for the P2 domain of CheA was determined by the cross-saturation experiments of [$^{2}$H, $^{15}$N] CheY and non-labeled CheA (Supplementary Fig. S8a). The V10-D12, R18-I20, N23-L24, D38, V40, F53, I55, D57, E67, V83-M85, K91, E93-A97, A99, A101-Y106, V108, F111, A113, K119, N121-E125, and L127 residues were affected by the irradiation (Supplementary Fig. S8b). Most of the affected residues, which are mapped in Supplementary Fig. S8c, were located at the binding site for the P1 domain of CheA (Fig. 6) and the binding site for the isolated P2 domain in the crystal structure of the isolated P2 domain-CheY complex (Supplementary Fig. S8d). These results indicated that the binding mode between the P2 domain of CheA and CheY is similar to that between the isolated P2 domain and CheY.

**Effects of modulation of the chemoreceptors on the temperature dependence of the CheY autophosphorylation reactions**

Ligand binding and methylation of the chemoreceptors affect the autophosphorylation rates of the CheA-chemoreceptor complexes, which determine the concentration of the phosphorylated CheA (CheA-P), and it is possible that the autophosphorylation rates are different among cells that express only one of the chemoreceptors. In the case of a low CheA-P concentration, the amount of phosphorylated CheY (CheY-P) decreases with increasing temperature, due to the decrease of the P1-CheY unbound state, in which autophosphorylation occurs (Supplementary Fig. S9a). The decrease of CheY-P leads to the motion to the higher temperature (warm-seeking behavior). In contrast, under the conditions with high CheA-P concentrations, CheY-P increases at higher temperatures, due to the increase of the P1-CheY bound state, in which phosphotransfer occurs. The decrease of CheY-P leads to the motion to the cold temperature (cold-seeking behavior). Although the aforementioned schemes are complicated by the phosphotransfer from CheA to CheB and the demethylation of the chemoreceptors by the phosphorylated CheB, our quantitative time-course calculation of the signaling demonstrated that the sign of the temperature-dependent change of the CheY-P concentration is perturbed by the attractant binding, and its sign can be switched by the complete methylation of the chemoreceptor

(Supplementary Fig. S9b). Therefore, the temperature-dependent shift of the dynamic domain arrangement of the CheA-CheY complex explains the effects of the chemoreceptors on the thermotaxis.

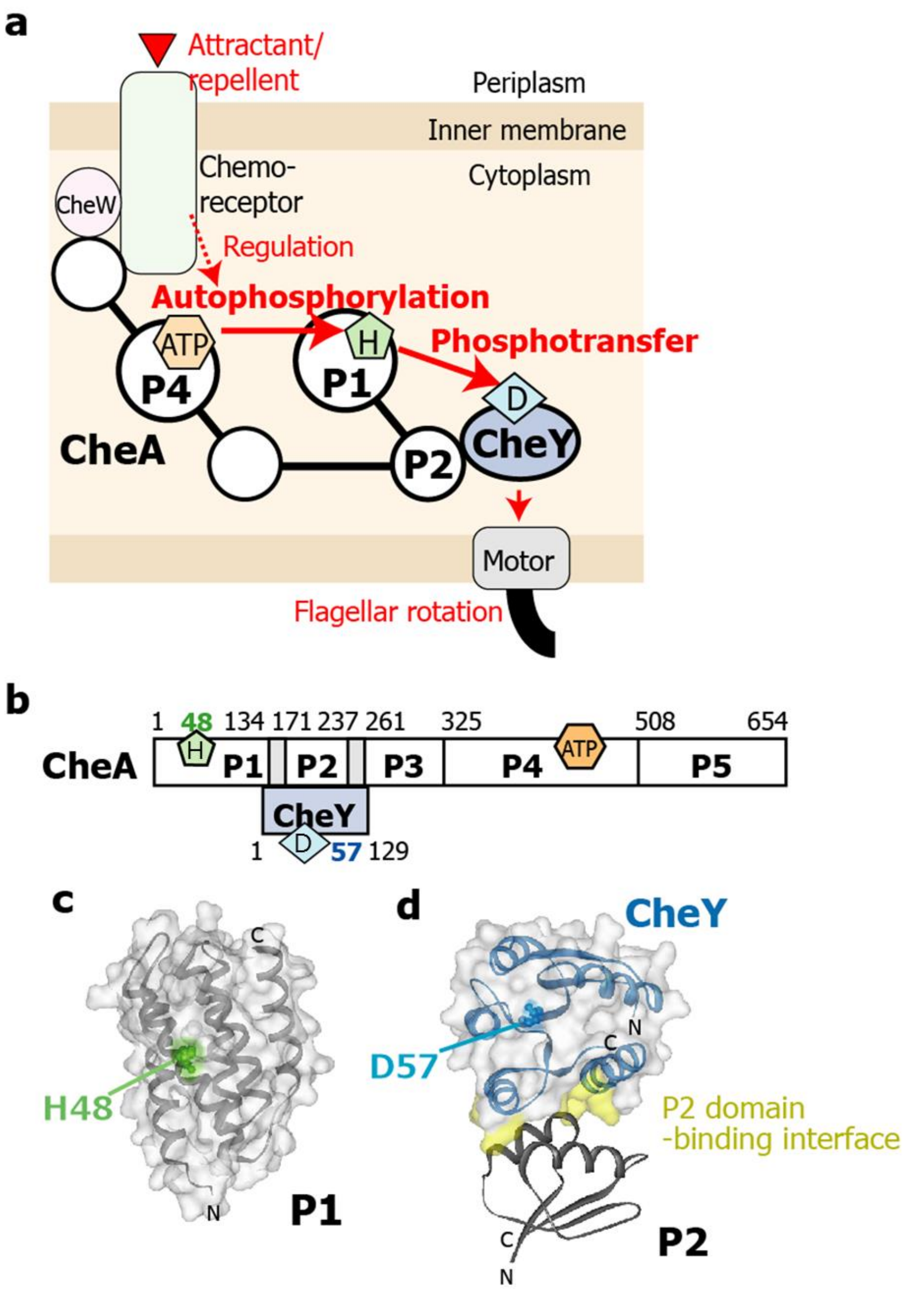


**Supplementary Fig. S1 CheA and CheY.** (a) Schematic diagram of the signal transduction in the bacterial chemotaxis system. (b) Domain organization of the CheA-CheY complex. The histidine residue in the P1 domain, the ATP-binding site in the P4 domain, and the aspartic acid residue in CheY, which are involved in the autophosphorylation and phosphotransfer reactions, are indicated. (c) and (d) Crystal

structures of the isolated P1 domain (c, PDB ID: 1I5N) and the isolated P2 domain-CheY complex (d, 1EAY). In (c) and (d), the surfaces of the P1 domain and CheY are transparent, and the ribbon diagrams are simultaneously displayed. The P2 domain-binding interface on CheY in the crystal structure is yellow.

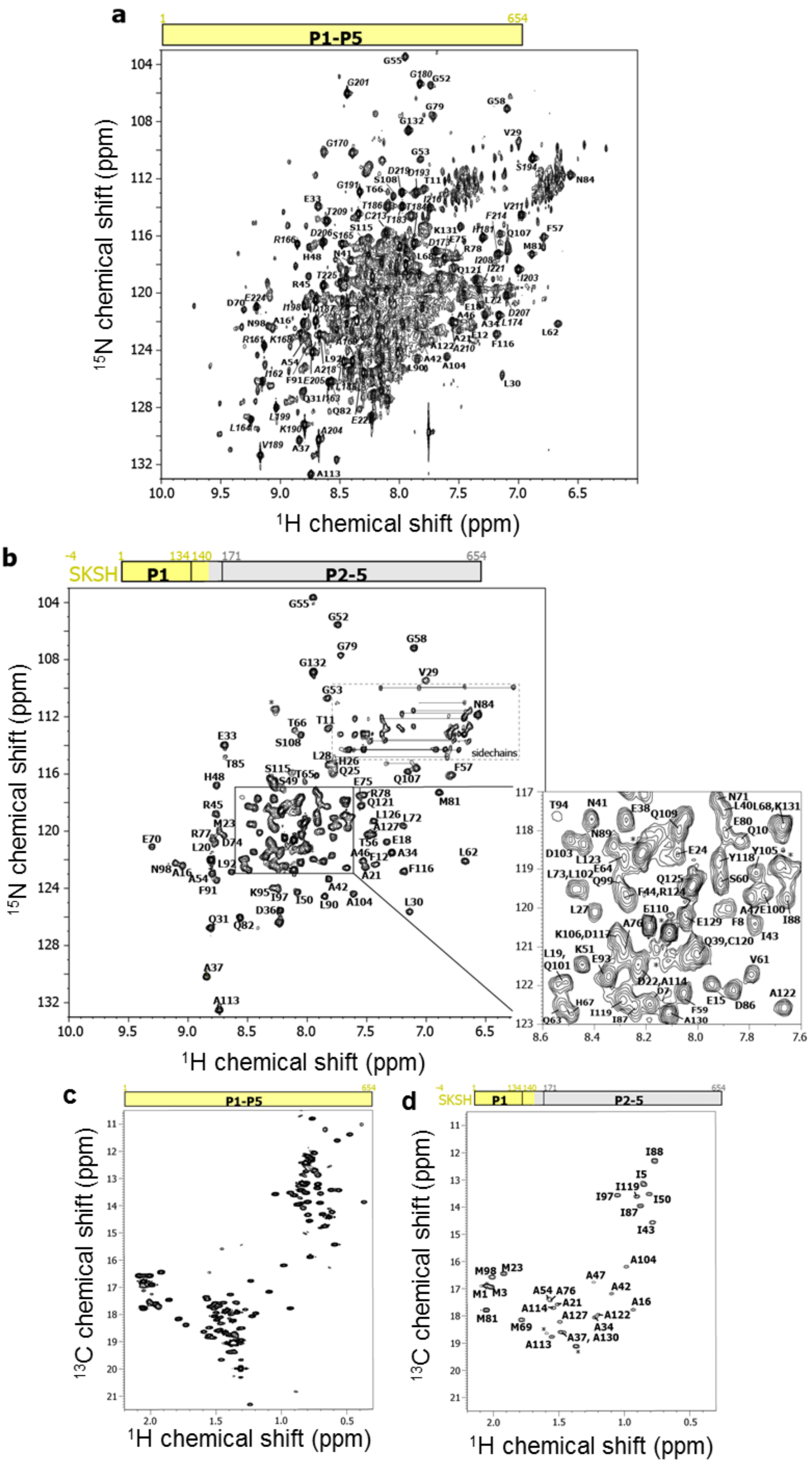


**Supplementary Fig. S2 $^{1}$H-$^{15}$N TROSY spectra of [$^{2}$H, $^{15}$N] CheA (a) and [$^{2}$H,$^{15}$N]-P1/P2–5 CheA (b), and $^{1}$H-$^{13}$C HMQC spectra of [u-$^{2}$H, Ileδ1-$^{13}$CH$_3$, Metε-$^{13}$CH$_3$,**

**Ala$\beta$-$^{13}CH_3$] CheA (c) and of [u-$^2H$, Ile$\delta$1-$^{13}CH_3$, Met$\varepsilon$-$^{13}CH_3$, Ala$\beta$-$^{13}CH_3$]-P1/P2–5 CheA (d).** Assignments of each signal are indicated. Resonances from the residues in the P1–P2 linker are marked with asterisks.

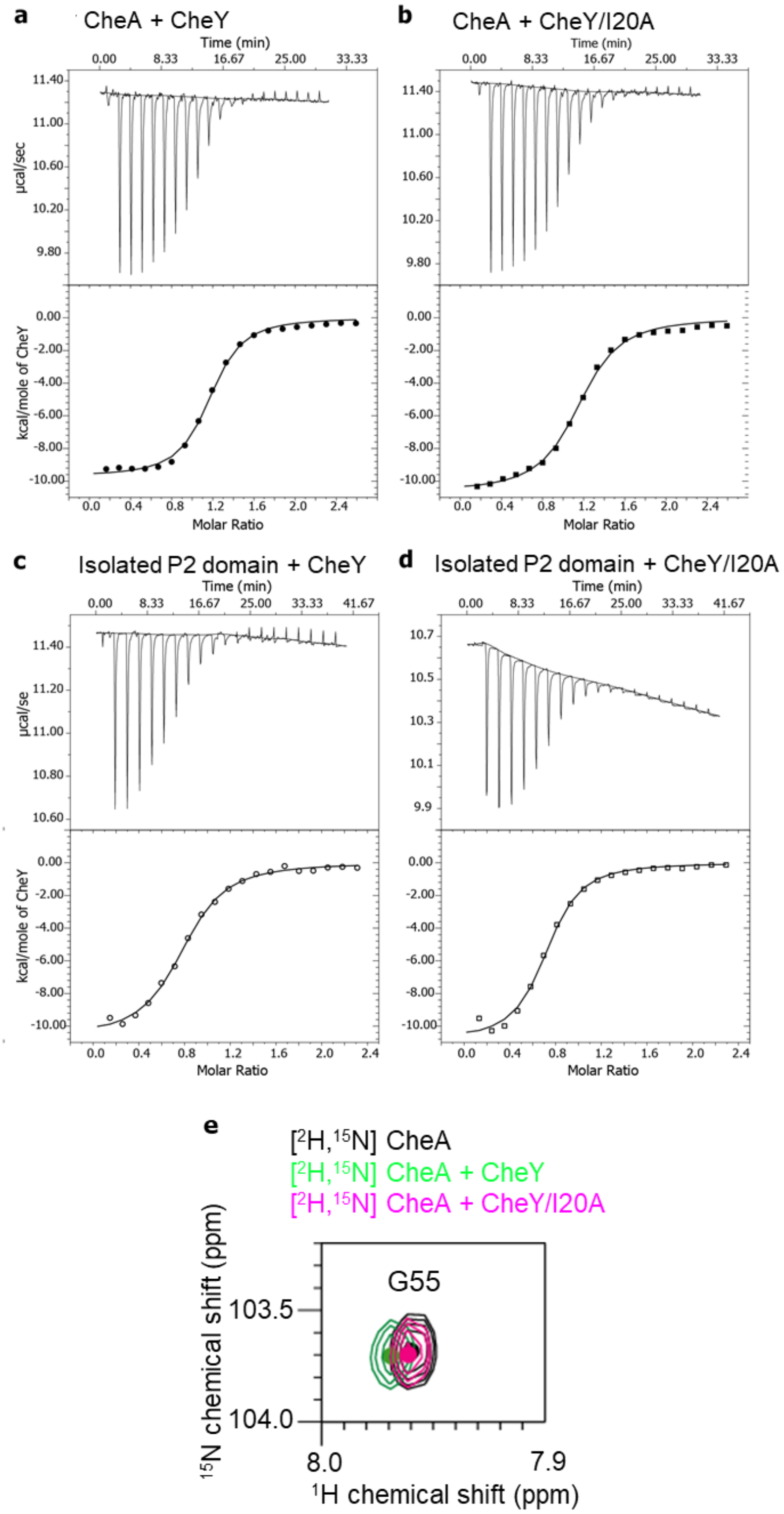


**Supplementary Fig. S3 Effect of the CheY I20A mutation on the CheA-CheY binding mode.** (a)-(d) Isothermal titration microcalorimetric analyses of the

interaction between CheA and CheY. Top, trace of the calorimetric titration with an initial injection of 0.2 uL, followed by aliquots (19 × 2 μL) of CheY (a and c) or CheY/I20A (b and d) into the cell containing CheA (a and b) or the isolated P2 domain (c and d). Bottom, integrated binding isotherm obtained by subtracting the isotherm of the heat of CheY dilution with the titration of CheY into the buffer with no protein. Solid lines show the best fit to a single-site model, resulting in the thermodynamic parameters in Supplementary Table S1. (e) Overlaid $^{1}H$-$^{15}N$ TROSY spectra of [$^{2}H$,$^{15}N$]-P1/P2–5 CheA in the absence of CheY (black) or in the presence of a stoichiometric amount of the wild type (green) or I20A mutant (magenta) of CheY. Only the regions with G55 resonances are shown, and the centers of the resonances are indicated with dots.

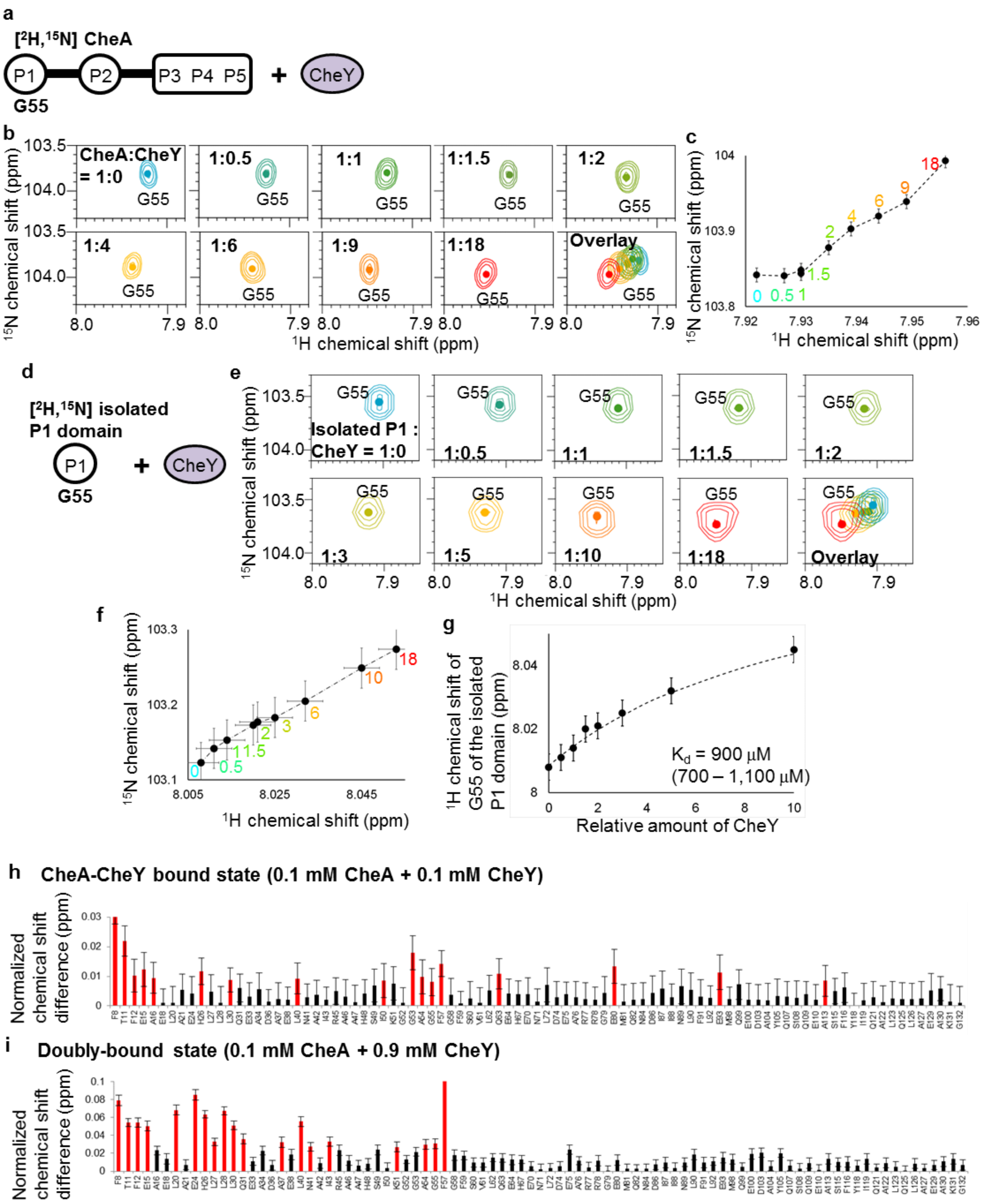


**Supplementary Fig. S4 Chemical shift changes of CheA and the isolated P1 domain upon binding to CheY.** (a-c) Chemical shift changes of G55 upon binding to CheY. (a) Schematic diagram of the experiment. (b) $^{1}$H-$^{15}$N TROSY spectra of [$^{2}$H, $^{15}$N]

CheA in the presence of various amounts of non-labeled CheY. (c) Plots of the $^1$H and $^{15}$N chemical shifts of the G55 resonances. (d-g) Chemical shift changes of the isolated P1 domain of CheA upon binding to CheY. (d) Schematic diagram of the experiment. (e) $^1$H-$^{15}$N TROSY spectra of the [$^2$H, $^{15}$N] isolated P1 domain in the presence of various amounts of non-labeled CheY. (f) Plots of the $^1$H and $^{15}$N chemical shifts of the G55 signals shown in (e). (g) Plots of the $^1$H chemical shifts as a function of the amount of CheY relative to the isolated P1 domain. (h-i) Chemical shift perturbations observed for the P1 domain of CheA upon the formation of the P1-CheY bound state and the doubly-bound state. (h) and (i) are plots of the normalized chemical shift differences of the resonances in the $^1$H-$^{15}$N TROSY spectra of the [$^2$H,$^{15}$N]-P1/P2–5 CheA between the free state and in the presence of 0.1 mM (h) or 0.9 mM (i) CheY. In (b) and (e), only the regions with G55 resonances are shown, and the centers are indicated with dots. The amount of CheY relative to CheA and the isolated P1 domain is displayed in the lower left corner of each spectrum, respectively, and the overlaid spectra are displayed in the lower-right panel. In (c) and (f), the relative amounts of CheY are indicated. Error bars in (c), (f), and (g) represent standard deviations of the chemical shifts of 100 synthetic time domain data from the *in situ* error analysis [84]. In (h) and (i), plots with normalized chemical shift differences larger than 0.008 ppm in (h) and 0.025 ppm in (i) are red. Normalized chemical shift differences, $\Delta\delta$, were calculated by the equation $\Delta\delta=[(\Delta\delta_{1H})^2+(\Delta\delta_{15N}/6.5)^2]^{0.5}$. The normalization factor (6.5) is the ratio of the standard deviation of the amide $^1$H and $^{15}$N chemical shifts, deposited in the Biological Magnetic Resonance Data Bank (http://www.bmrb.wisc.edu/) [85]. The error values were calculated by the formula $(\Delta\delta_{1H}\cdot R_{1H} + \Delta\delta_{15N}\cdot R_{15N}/6.5)/\Delta\delta$, where $R_{1H}$ and $R_{15N}$ are the standard deviations of the

$^{1}H$ and $^{15}N$ chemical shifts of 100 synthetic time domain data from the *in situ* error analysis [84], respectively.

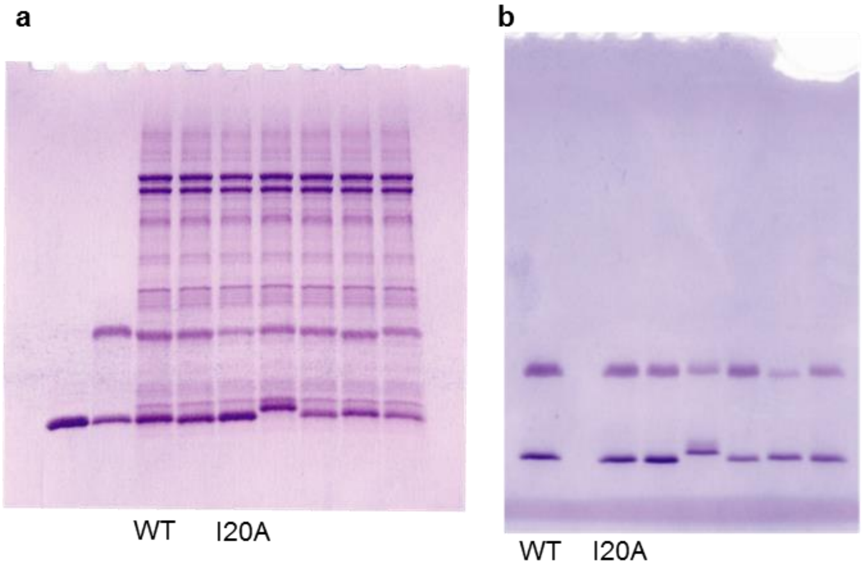


**Supplementary Fig. S5 Full-size gel images of Fig. 1h (a) and Fig. 1i (b).**

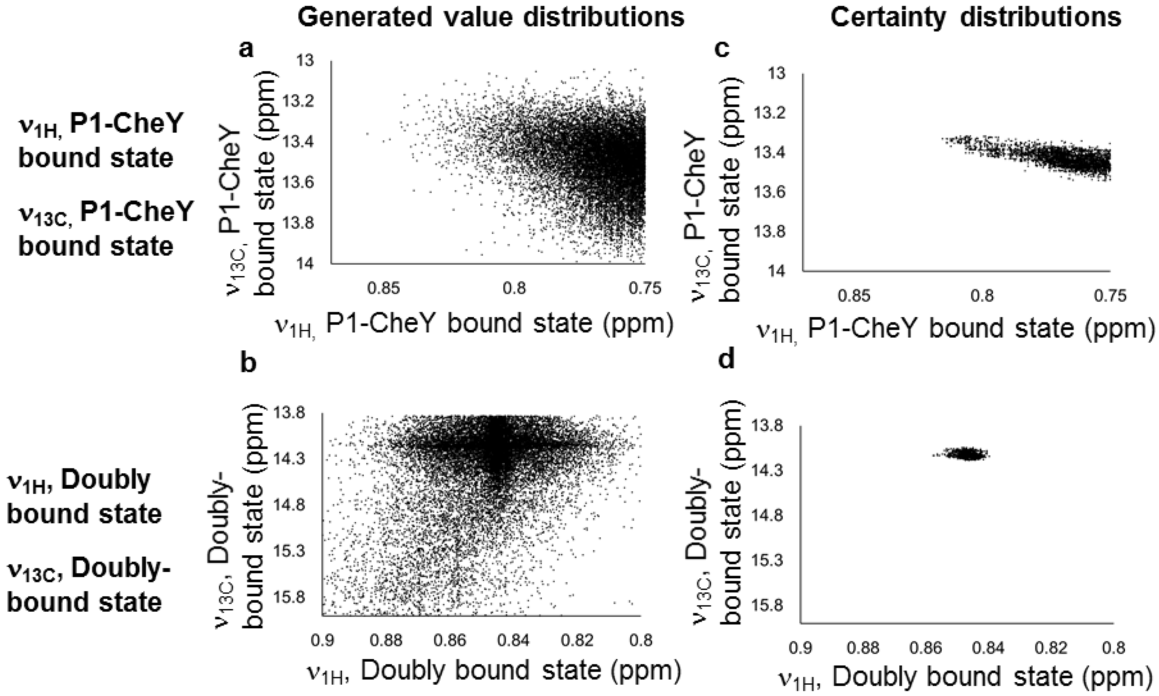


**Supplementary Fig. S6 Generated value distributions and certainty distributions of the chemical shifts of the P1-CheY bound state and the doubly-bound state.** (a)-(b) Plots of the generated $^{1}$H chemical shifts of the P1-CheY bound state (a) and the doubly-bound state (b) against their $^{13}$C chemical shifts. (c)-(d) Certainty distributions of the $^{1}$H chemical shifts of the P1-CheY bound state (c) and the doubly-bound state (d) against their $^{13}$C chemical shifts. In (c), and (d), data during the burn-in period were excluded from the plots.

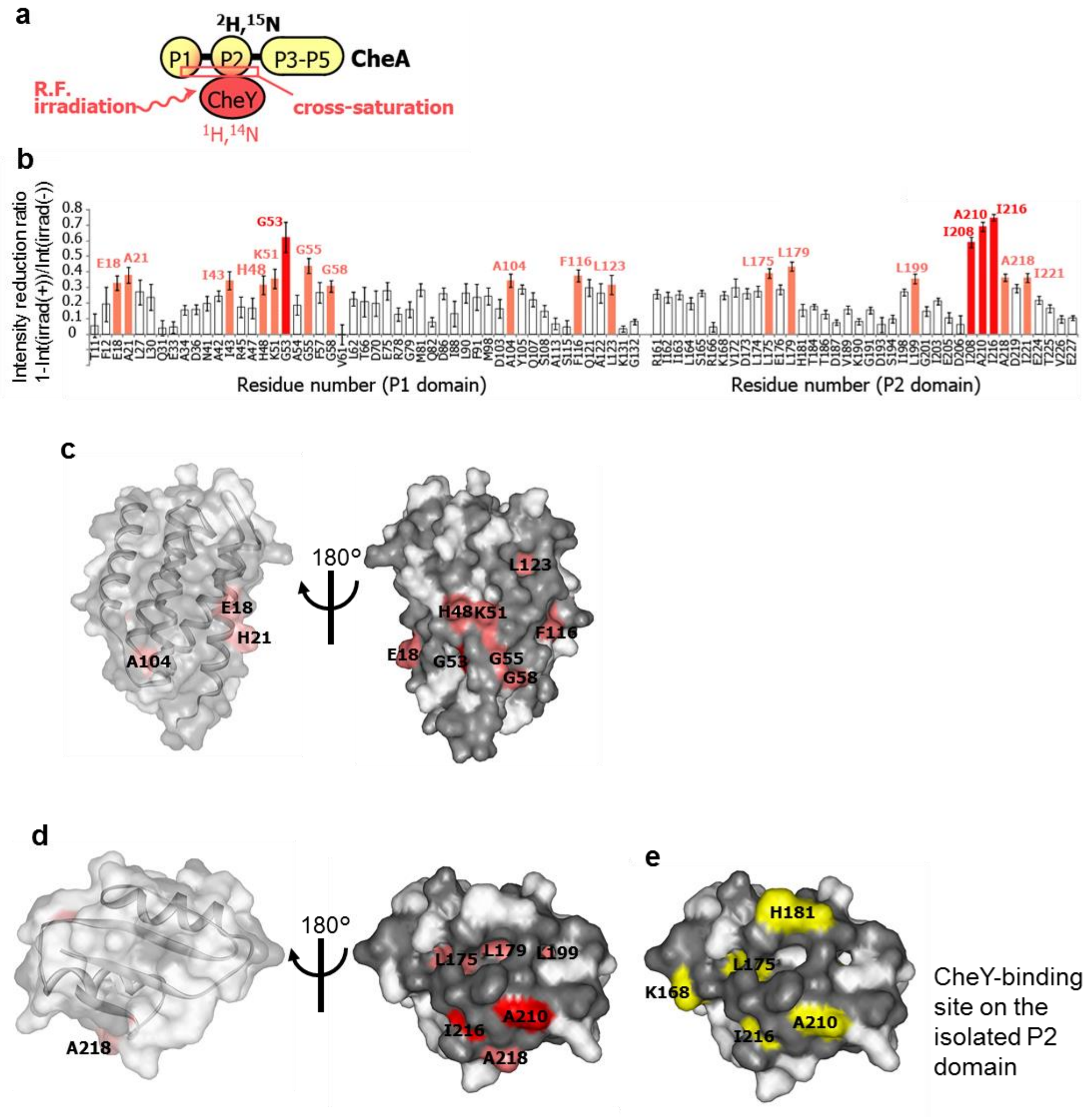


**Supplementary Fig. S7 CheY-binding interface on CheA revealed by cross-saturation experiments.** (a) Schematic diagram of the experiment. (b) Plots of the reduction ratios of the signal intensities originating from the amide groups, with and without presaturation. Red and light orange plots represent the residues with signal intensity reduction ratios > 0.5 and within the 0.3- 0.5 range, and are labeled. The residues with reduction ratios < 0.3 are white. The error bars represent the root sum square of the reciprocal of the signal-to-noise ratio of the resonances with and without

irradiation. (c) and (d) Mapping of the residues on the P1 (c) and P2 (d) domains of CheA affected by the irradiation in the cross-saturation experiments (PDB IDs: 1I5N and 1EAY). The residues with signal intensity reduction ratios > 0.5 and within the 0.3-0.5 range are colored red and light orange, respectively. Proline residues and the residues with intensity reductions that were not determined, because of low signal intensity or spectral overlap, are gray. In the view on the left, the surfaces of the P1 and P2 domains are transparent, and the ribbon diagrams are simultaneously displayed. (e) CheY binding interface on the isolated P2 domain in the crystal structure of the isolated P2 domain-CheY complex (PDB ID: 1EAY). The labeling and coloring schemes are the same as in Fig. 6f. The molecular diagrams were generated with Web Lab Viewer Pro (Molecular Simulations, Inc.).

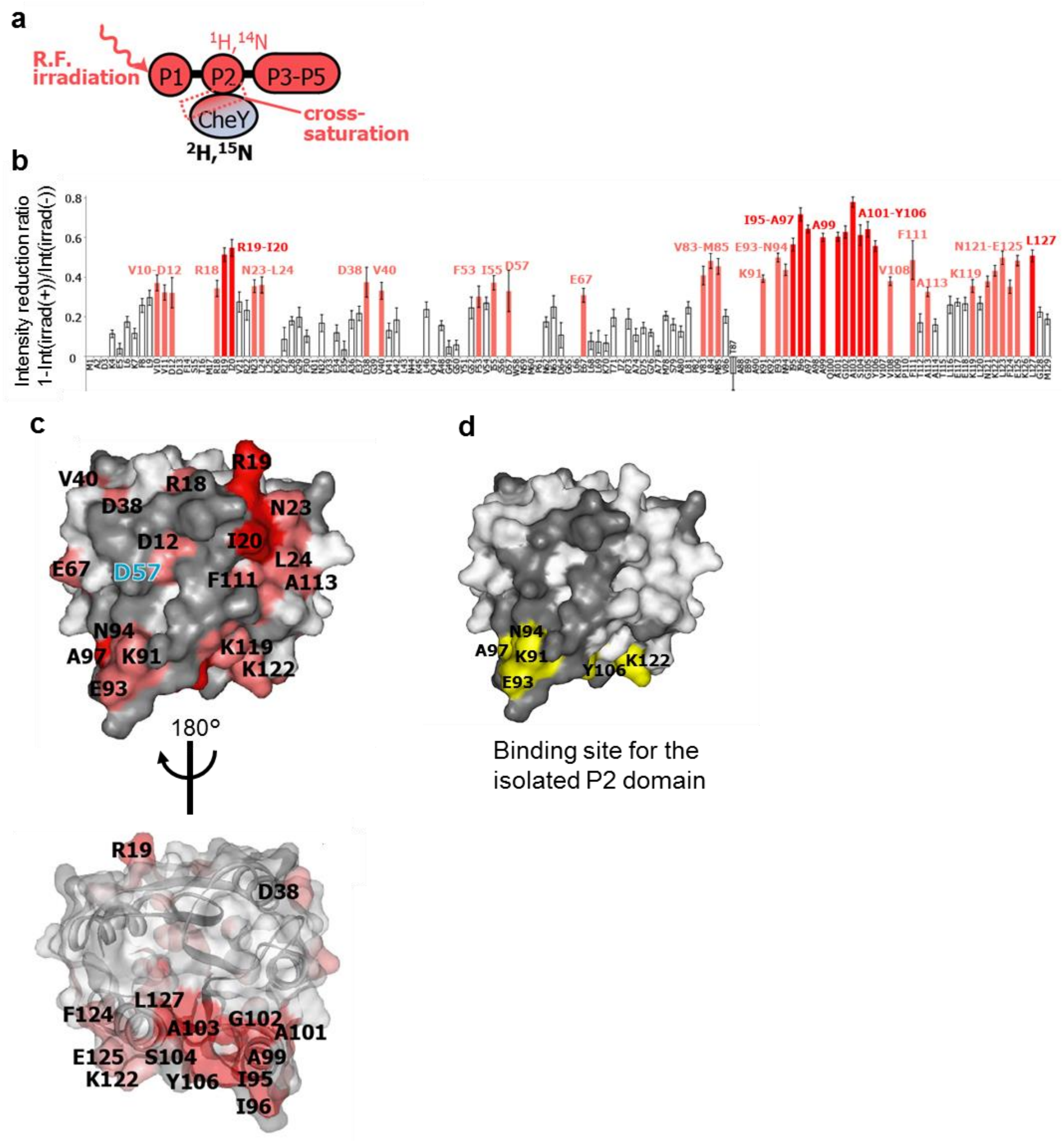


**Supplementary Fig. S8 CheA-binding interface on CheY revealed by cross-saturation experiments.** (a) Schematic diagram of the experiment. (b) Plots of the reduction ratios of the signal intensities originating from the amide groups, with and without presaturation. Red and light orange plots represent the residues with signal intensity reduction ratios > 0.5 and within the 0.3- 0.5 range, and are labeled. The residues with reduction ratios < 0.3 are white. The error bars represent the root sum

square of the reciprocal of the signal-to-noise ratio of the resonances with and without irradiation. (c) Mapping of the residues on CheY affected by the irradiation in the cross-saturation experiments (PDB ID: 1EAY). The residues with signal intensity reduction ratios > 0.5 and within the 0.3-0.5 range are colored red and light orange, respectively. Proline residues and the residues with intensity reductions that were not determined, because of low signal intensity or spectral overlap, are gray. In the bottom view, the surface of CheY is transparent, and the ribbon diagram is simultaneously displayed. (d) Isolated P2 domain-binding interface on CheY in the crystal structure of the isolated P2 domain-CheY complex (PDB ID: 1EAY). The coloring and labeling schemes are the same as in Fig. 6f. The molecular diagrams were generated with Web Lab Viewer Pro (Molecular Simulations, Inc.).

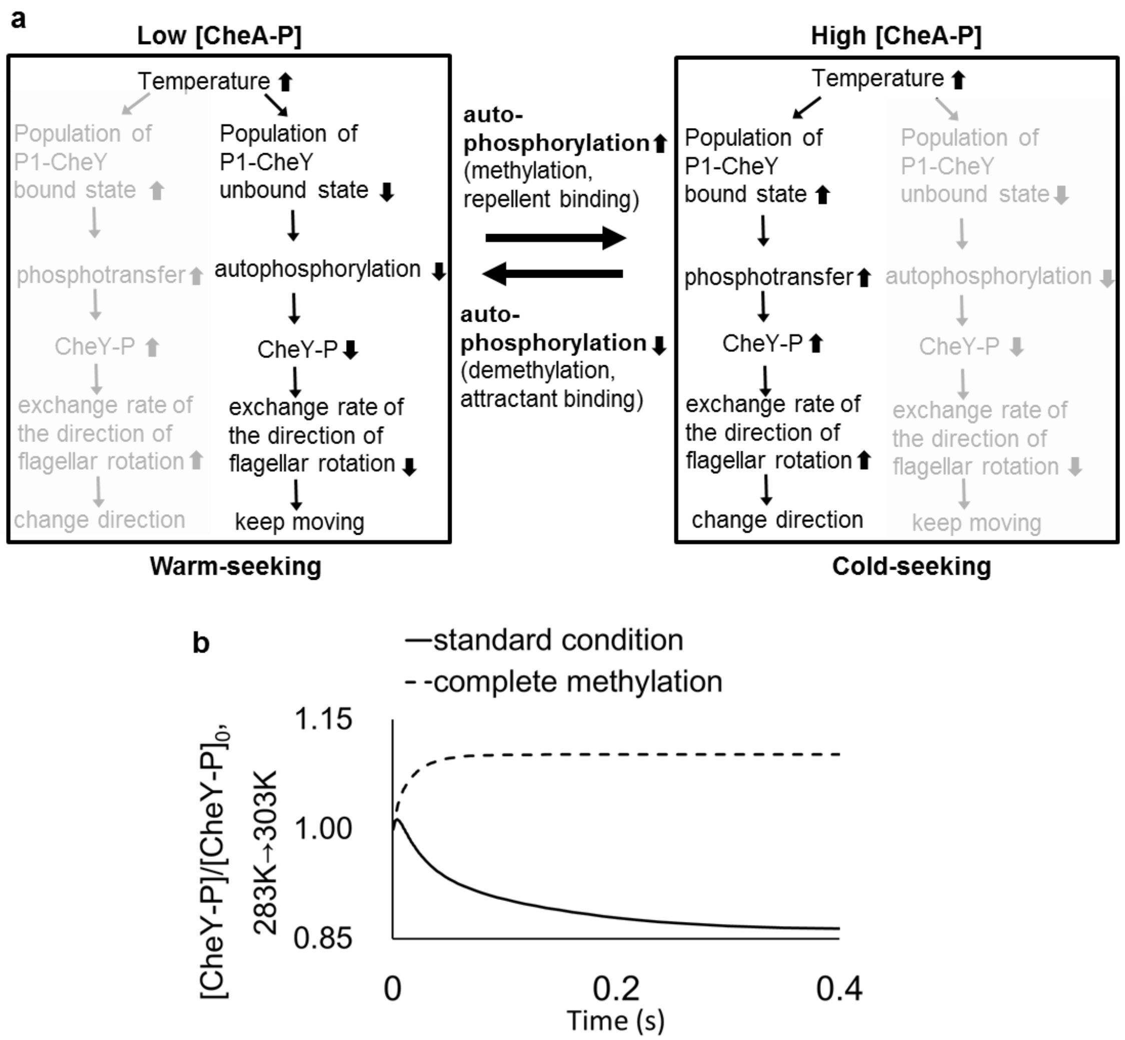


**Supplementary Fig. S9 Effects of the modulation of the chemoreceptors on the temperature dependence of the CheY phosphorylation reactions.** (a) Scheme of the effects of the modulation of the chemoreceptors on the temperature dependence of the CheY phosphorylation reactions. (b) Calculated time-course of the amount of phosphorylated CheY upon increasing the temperature from 283 K to 303 K. Solid and dashed lines are the data obtained under the standard conditions and the conditions where the chemoreceptor was fully methylated, respectively.

**Supplementary Table S1. Stoichiometry N, dissociation constants $K_d$, enthalpy changes ΔH, and entropy changes ΔS of the CheA-CheY interactions determined by the ITC experiments (Supplementary Fig. S3a-d).**

| Cell | Syringe | N | $K_d$ (μM) | ΔH (kcal/mol) | ΔS (kcal/mol/deg) | # of experiments |
|---|---|---|---|---|---|---|
| CheA | CheY | 1.1 ± 0.1 | 1.6 ± 0.4* | -10.5 ± 0.5 | -6.7 ± 1.8 | 3 |
| CheA | CheY/I20A | 1.1 ± 0.1 | 2.6 ± 0.7* | -10.7 ± 0.3 | -9.7 ± 0.7 | 4 |
| Isolated P2 domain | CheY | 0.75 ± 0.03 | 2.6 ± 0.2 | -10.8 ± 0.1 | -10.2 ± 0.6 | 2 |
| Isolated P2 domain | CheY/I20A | 0.74 ± 0.07 | 2.2 ± 0.6 | -10.8 ± 0.6 | -9.8 ± 1.7 | 3 |

Data represent the mean ± SE mean of two, three, or four separate experiments.

*$P < 0.05$, unpaired one-tailed Student t test.

**Supplementary Table S2. Parameters of quantitative calculations of bacterial chemotaxis signaling[79-82,86].**

| | Description | Value |
|---|---|---|
| $[T]_{tot}$ | Total chemoreceptor concentration | 5 nM |
| $[A]_{tot}$ | Total CheA concentration | 5 μM |
| $[Y]_{tot}$ | Total CheY concentration | 17.9 μM |
| $[B]_{tot}$ | Total CheB concentration | 2 μM |
| $[M]_{tot}$ | Total FliM concentration | 5.8 μM |
| $[R]$ | CheR concentration | 0.235 μM |
| $k_{auto}$ | Rate of CheA autophosphorylation | 8 $s^{-1}$ |
| $k_{ApY}$ | Rate of phosphotransfer from CheA to CheY | $10^8$ $M^{-1}s^{-1}$ |
| $k_{ApB}$ | Rate of phosphotransfer from CheA to CheB | $5\times10^6$ $M^{-1}s^{-1}$ |
| $k_{Yp}$ | Rate of autodephosphorylation of CheY | 0.1 $s^{-1}$ |
| $k_{YpZ}$ | Rate of dephosphorylation of CheY by CheZ | 30 $s^{-1}$ |
| $k_{YpM}$ | Rate of association of phosphorylated CheY and FliM | $5\times10^6$ $M^{-1}s^{-1}$ |
| $k_{MYp}$ | Rate of dissociation of phosphorylated CheY-FliM complex | 19 $s^{-1}$ |
| $k_{Bp}$ | Rate of autodephosphorylation of CheB | 1 $s^{-1}$ |
| $k_b$ | $k_{cat}$ of the demethylation by CheB | 0.5 $s^{-1}$ |
| $K_B$ | Michaelis constant of the demethylation by CheB | 5.5 nM |
| $k_r$ | $k_{cat}$ of the methylation by CheR | 0.255 $s^{-1}$ |

| | | |
|---|---|---|
| $K_R$ | Michaelis constant of the methylation by CheR | 0.251 nM |
| $K_L$ | Dissociation constant of chemoreceptor and attractant | 10 μM |
| $p_0$ | Active probability of free chemoreceptor without methylation | 0 |
| $p_1$ | Active probability of free chemoreceptor with one methylated residue | 0.1 |
| $p_2$ | Active probability of free chemoreceptor with two methylated residues | 0.5 |
| $p_3$ | Active probability of free chemoreceptor with three methylated residues | 0.75 |
| $p_4$ | Active probability of free chemoreceptor with four methylated residues | 1 |
| $p_0^L$ | Active probability of chemoreceptor-attractant complex without methylation | 0 |
| $p_1^L$ | Active probability of chemoreceptor-attractant complex without one methylated residue | 0 |
| $p_2^L$ | Active probability of chemoreceptor-attractant complex without two methylated residues | 0.1 |
| $p_3^L$ | Active probability of chemoreceptor-attractant complex without three methylated residues | 0.5 |
| $p_4^L$ | Active probability of chemoreceptor-attractant complex without four methylated residues | 1 |
